# Diet and landscape characteristics drive spatial patterns of mercury accumulation in a high-latitude terrestrial carnivore

**DOI:** 10.1371/journal.pone.0285826

**Published:** 2023-05-15

**Authors:** Inés Peraza, John Chételat, Murray Richardson, Thomas S. Jung, Malik Awan, Steve Baryluk, Ashu Dastoor, William Harrower, Piia M. Kukka, Christine McClelland, Garth Mowat, Nicolas Pelletier, Christine Rodford, Andrei Ryjkov

**Affiliations:** 1 Geography and Environmental Studies, Carleton University, Ottawa, Ontario, Canada; 2 Environment and Climate Change Canada, National Wildlife Research Centre, Ottawa, Ontario, Canada; 3 Department of Environment, Government of Yukon, Whitehorse, Yukon, Canada; 4 Department of Renewable Resources, University of Alberta, Edmonton, Alberta, Canada; 5 Department of Environment, Government of Nunavut, Igloolik, Nunavut, Canada; 6 Environment and Natural Resources, Government of the Northwest Territories, Inuvik, Northwest Territories, Canada; 7 Environment and Climate Change Canada, Air Quality Research Division, Dorval, Quebec, Canada; 8 Forest and Conservation Sciences, University of British Columbia, Vancouver, British Columbia, Canada; 9 Ministry of Forests, British Columbia Government, Nelson, British Columbia, Canada; 10 Department of Earth, Environmental and Geographic Sciences, University of British Columbia, Kelowna, British Columbia, Canada; Indian Institute of Technology Hyderabad, INDIA

## Abstract

Limited information exists on mercury concentrations and environmental drivers of mercury bioaccumulation in high latitude terrestrial carnivores. Spatial patterns of mercury concentrations in wolverine (*Gulo gulo*, n = 419) were assessed across a 1,600,000 km^2^ study area in relation to landscape, climate, diet and biological factors in Arctic and boreal biomes of western Canada. Hydrogen stable isotope ratios were measured in wolverine hair from a subset of 80 animals to assess the spatial scale for characterizing environmental conditions of their habitat. Habitat characteristics were determined using GIS methods and raster datasets at two scales, the collection location point and a 150 km radius buffer, which was selected based on results of a correlation analysis between hydrogen stable isotopes in precipitation and wolverine hair. Total mercury concentrations in wolverine muscle ranged >2 orders of magnitude from 0.01 to 5.72 μg/g dry weight and varied geographically, with the highest concentrations in the Northwest Territories followed by Nunavut and Yukon. Regression models at both spatial scales indicated diet (based on nitrogen stable isotope ratios) was the strongest explanatory variable of mercury concentrations in wolverine, with smaller though statistically significant contributions from landscape variables (soil organic carbon, percent cover of wet area, percent cover of perennial snow-ice) and distance to the Arctic Ocean coast. The carbon and nitrogen stable isotope ratios of wolverine muscle suggested greater mercury bioaccumulation could be associated with feeding on marine biota in coastal habitats. Landscape variables identified in the modelling may reflect habitat conditions which support enhanced methylmercury transfer to terrestrial biota. Spatially-explicit estimates of wet atmospheric deposition were positively correlated with wolverine mercury concentrations but this variable was not selected in the final regression models. These landscape patterns provide a basis for further research on underlying processes enhancing methylmercury uptake in high latitude terrestrial food webs.

## Introduction

Methylmercury (MeHg), the organic form of mercury (Hg), is a neurotoxin that has been linked to adverse hormonal changes, reproduction and motor skill impairment in fish, birds and mammals [1–4]. Mercury contamination has been extensively studied in aquatic ecosystems [5, 6], but environmental controls on Hg bioaccumulation in terrestrial ecosystems and wildlife remain poorly understood, particularly in the Arctic [7–9]. Herein, we use a broad geographic definition of the Arctic, which includes subarctic and Arctic regions [7, 8]. Recent studies have addressed Hg exposure in terrestrial Arctic wildlife, mostly caribou (*Rangifer tarandus*) [8, 10] and some studies on predators, such as polar bear (*Ursus maritimus*), Arctic fox (*Vulpes lagopus*) and gray wolf (*Canis lupus*) [11–15]. However, further information is needed on the mechanisms and factors driving inorganic Hg methylation in terrestrial habitats, the routes of Hg exposure to Arctic food webs and the environmental factors controlling Hg concentrations in Arctic terrestrial wildlife [8, 9].

Inorganic Hg in the atmosphere is transported through air to higher latitudes where it is deposited on aquatic and terrestrial ecosystems [16–18]. Snow, ice and soils on land are key repositories of atmospheric Hg deposition in the Arctic, which could be released due to climate warming [8, 19–21]. In Arctic regions, Hg deposition to snow and ice is enhanced during polar spring by Atmospheric Mercury Depletion Events (AMDEs), which occur in marine and coastal areas [21–23]. During AMDEs, reactive halogens drive photo-oxidation of gaseous elemental Hg (Hg^0^) to gaseous oxidized Hg [Hg(II)], which can bond with aerosol to form Hg(II) particulate Hg [Hg_(p)_] [23]. Although most of the Hg(II) deposited in the snowpack is photo-reduced and emitted back to the atmosphere as Hg^0^ within days, a fraction of the Hg can be retained in the snowpack [18, 22, 23]. Since AMDEs add bioavailable Hg to snow and tundra soils, it may be a significant path of atmospheric Hg transfer to Arctic biota [9]. However, it is unclear how important Hg cycling and methylation in Arctic snow, ice, glaciers, and permafrost soils is to the build-up of Hg in the terrestrial Arctic environment [8]. In the Arctic, vegetation plays an important role in terrestrial Hg^0^ uptake. For instance, during the snow-free season, uptake of Hg^0^ by tundra plants can represent up to 70% of the total atmospheric deposition leading to high Hg levels in Arctic soils [24, 25]. Concentrations of total Hg (MeHg + inorganic Hg, [THg]) in Arctic vegetation tends to be higher in tundra biomes than forest biomes and greater in non-vascular plants (e.g., terrestrial lichens and mosses) than in vascular plants [8, 9, 26, 27]. In the Canadian High Arctic, higher THg and MeHg concentrations were found in lichens from coastal areas compared with inland [27]. Mercury accumulation in lichens and plants is a key uptake route for terrestrial food webs leading to dietary exposure to herbivores and their predators. Consumers bioaccumulate dietary MeHg in tissues including liver, muscle and brain, and concentrations tend to increase as an animal ages [28].

Naturally occurring stable isotopes have been extensively used in biology and ecology to identify environmental patterns and processes [29]. For example, the stable isotope composition of wildlife tissues can be valuable tracers of feeding ecology (e.g., trophic interactions and diet) and animal movement or origin [30–33]. Stable isotope ratios of carbon (δ^13^C) have been used to identify the origin of carbon utilized by consumers and can provide insight into contaminant exposure via marine, terrestrial and aquatic feeding [15, 34, 35]. Stable isotope ratios of nitrogen (δ^15^N) increase generally 2.5–5‰ per trophic level [36–38] and have been used to estimate organism trophic position [11, 34]. Higher Hg concentrations have been found in wildlife at a higher trophic position due to biomagnification [11, 28]. Hydrogen stable isotope ratios (δD) have been widely used to understand migratory connectivity, dispersal movements, and geographic origins of aquatic and terrestrial species [31, 32, 38]. Ratios of H in metabolically inert keratinous tissues (e.g., hair, feather) of wildlife can provide information on animal movements and origin because the hydrogen isotope signature (reflecting food and water intake) is related to broad-scale geographic patterns of δD in precipitation (δD_precip_). Recent innovative approaches are emerging to assess Hg concentrations and exposure pathways in wildlife using δD in keratinous tissues [39].

The wolverine (*Gulo gulo*) is a wide-ranging carnivore that is broadly distributed throughout Arctic and boreal biomes in North America and the Holarctic northern hemisphere [40, 41]. In Canada, wolverine are listed as a species at risk [41] and there is conservation concern for the species in many parts of its range especially the lower 48 states in the USA. Wolverines are both predators and scavengers and occur at very low densities and consequentially huge ranges [42]. They are capable of killing large prey such as caribou [43] but also regularly kill smaller prey such as hares (*Lepus americanus*) and marmots (*Marmota* spp.) [42]. Low densities, large spatial requirements, low reproductive rate and long term declines of prey abundance may contribute to wolverine vulnerability [44–48]. Lastly, wolverine avoid areas occupied or used by people [49] which can lead to lower densities near human dominated landscapes [48]. Consequently, wolverines are potential indicators of ecosystem health and habitat change [46] and our study aimed to investigate Hg bioaccumulation in wolverines in northern Canada.

There is limited information on Hg bioaccumulation in wolverine [50–52]. Our main objectives were to assess and model the spatial patterns of THg accumulation in wolverine across northern Canada, and to identify landscape, climate, dietary and biological factors associated with those patterns. We first evaluated the application of δD in wolverine hair as an aid to decide the appropriate spatial scale for the analysis of environmental influences on wolverine THg concentration. Then we characterized spatial patterns of THg concentration in wolverine in northern Canada. Finally, we evaluated environmental factors that may influence THg concentrations, including Hg deposition rates, climatic conditions, and landscape characteristics. To our knowledge this is the first dataset on muscle concentrations of THg in the species. Therefore, this study generated baseline information with a broad geographic scope on THg concentrations in wolverine in high-latitude terrestrial ecosystems.

## Materials and methods

### Ethics statement

Animal samples used in this study were collected over a 13-year period (2005–2018) from wolverine carcasses submitted to wildlife management agencies by either licensed trappers operating under individual licenses issued by those agencies to harvest those animals or by Inuit beneficiaries with harvesting rights. The use of samples from carcasses of harvested animals was conducted under the full authority of provincial and territorial wildlife acts in British Columbia, Northwest Territories, Nunavut, and the Yukon, with permits when required for government employees. In Nunavut, work was conducted under Nunavut Wildlife Research permits WL 2010–011, WL 2011–011, WL 2012–037, and WL 2014–017.

### Wolverine tissue collection

**Fig 1:**
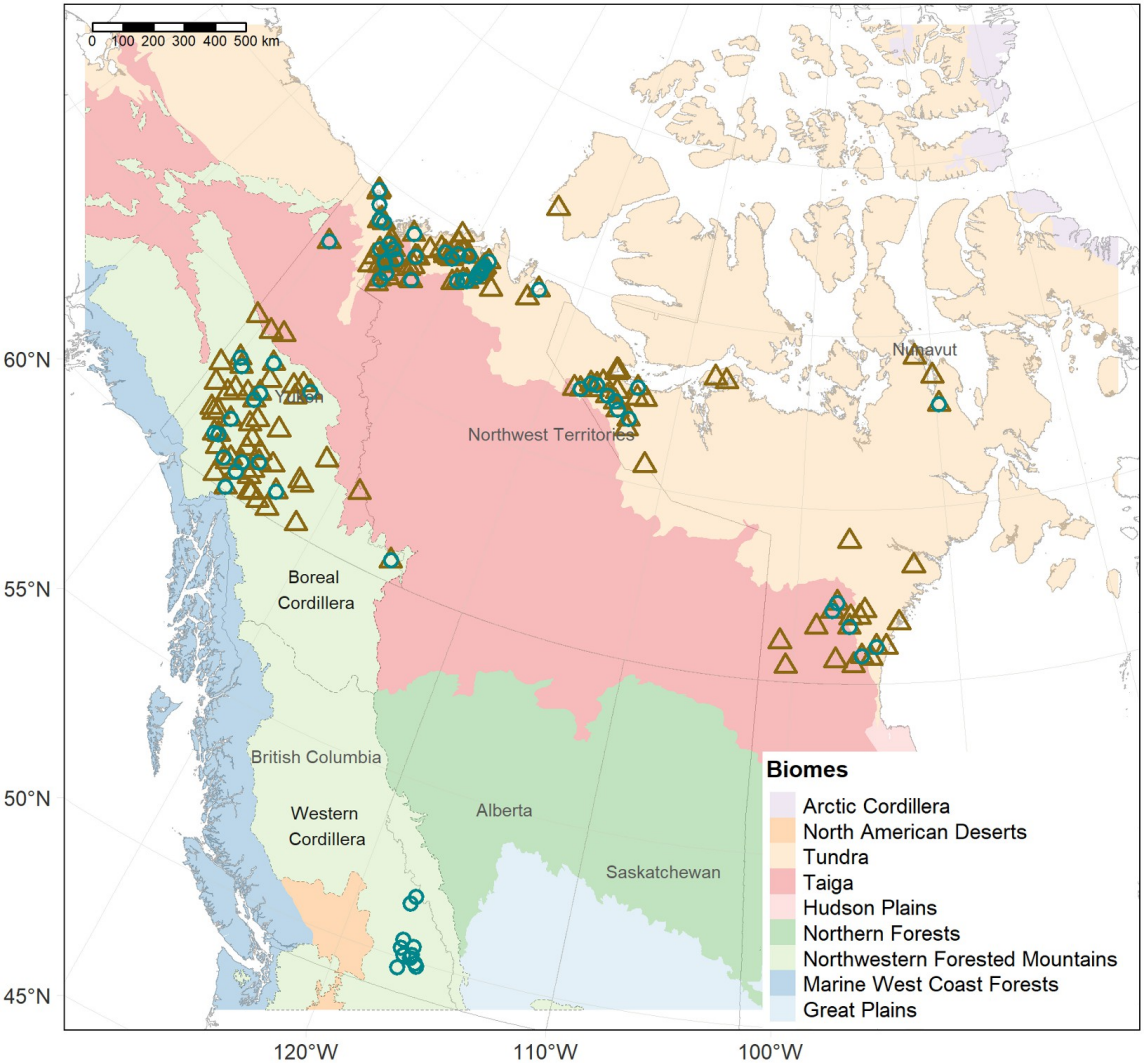
Location of wolverine collection sites in Canada. Brown triangles represent muscle samples (n = 419) and the green circles hair samples (n = 80) from wolverine (*Gulo gulo*). The biomes correspond to the North American ecological regions, level I [54]. Northwestern Forested Mountains was subdivided in Boreal Cordillera and Western Cordillera (ecological region level II).

Wolverine muscle and hair samples were obtained from trapper submitted carcasses from Canada’s three northern territories and from hair snags from the province of British Columbia (BC) (Fig 1). Carcass collection programs were conducted in Yukon (YT), the Northwest Territories (NT) and Nunavut (NU) [53]. In addition to sampling Arctic and subarctic regions of northwestern Canada above 60° N latitude, hair samples were obtained via hair snags from a wolverine study in the interior of BC [48]. One hair and/or muscle sample was obtained per individual animal.

Where possible, collection date, location name, latitude, longitude, sex, and age were recorded for each sample. The precision of collection location may have varied for different animals. In certain areas, the location coordinates may refer to a more general area (e.g., trap lines). The harvest seasons for hair samples ranged from 2007–2008 to 2017–2018 and from 2005–2006 to 2017–2018 for muscle. Animals >2 years old were classified as adults and ≤ 2 years old as sub-adults, based on the minimum reported ages of reproductive maturity for female wolverines [53, 55, 56].

### Laboratory analyses

Whole hairs were obtained from 80 wolverines (31 females, 49 males) and were analyzed for δD. The hair collection locations were broadly distributed including additional samples from BC to maximize the range of hair δD values (Fig 1). Hair was pre-washed with a 2:1 chloroform: methanol solution to remove oils consistent with previous methods [57]. The analysis for δD was performed on an Isotope Ratio Mass Spectrometer at the Ján Veizer Stable Isotope Laboratory at the University of Ottawa, Canada. The H isotope results were reported for non-exchangeable H, in delta notation (δ), in per-mil units (‰) and normalized on the standard Vienna Standard Mean Ocean Water (VSMOW) scale. The analytic error reported is +/- 2‰, which according to Wunder & Norris [58] is common in other studies. The average H exchange ratio for all the wolverine samples was 5.2% using previous methods [59].

Total Hg concentration was measured in the hind muscle of 419 wolverine (147 females, 271 males). Most of the muscle samples were from YT (n = 316). Smaller samples sizes were available for the NT (n = 59) and NU (n = 44) (Fig 1). Freeze-dried and homogenized muscle samples were analyzed on a dry weight (dw) basis with a Direct Mercury Analyzer (Milestone Inc, Italy) at the National Wildlife Research Centre, Ottawa, Canada. Quality control procedures included measurement of blanks (< 0.07 ng Hg) and duplicates of samples (mean relative standard deviation 4.4%, n = 144). The following certified reference materials were used to confirm accuracy of the method: IAEA-436 (mean recovery 99.3%, n = 47), and TORT-3 (mean recovery 102.4%, n = 157). Muscle was also analyzed for δ^15^N and δ^13^C at the Cornell Stable Isotope Laboratory (Cornell University, Ithaca, NY). Stable isotope ratios were expressed in δ (‰) deviation from standards of atmospheric N_2_ gas for δ^15^N and Vienna PeeDee Belemnite for δ^13^C.

### Landscape and climate data

Environmental variables were chosen based on prior scientific knowledge of factors that could influence THg concentration in terrestrial wildlife and ecosystems. Table 1 describes the landscape and climate variables identified in the literature and selected for statistical analysis.

**Table 1:** Landscape and climate factors considered for statistical analysis.

Factors	Rationale	Sources
Net and wet Hg deposition	Positive relationship between atmospheric Hg deposition with Hg concentration in wildlife	[60]
Precipitation	Atmospheric deposition of Hg is largely through precipitation Precipitation is one of the major controls of soil Hg mobilization	[20, 21]
Temperature	Temperature may be important for predicting Hg concentrations in aquatic environments Warmer temperatures potentially release Hg within the snowpack and perennial ice Warmer Arctic temperatures can enhance MeHg production	[8, 61]
Land cover	Some types of vegetation cover may contribute to Hg loadings in terrestrial ecosystems because of the role of vegetation in the uptake of Hg from air Higher THg concentrations have been reported in the Arctic tundra than in boreal forests Higher THg were reported in Arctic non-vascular plants (e.g., terrestrial lichens and mosses) than in Arctic vascular plants Water-logged terrestrial soils are potential production sites of MeHg in the Arctic	[8, 26, 62]
Elevation	Deposition of Hg can be related to elevation gradients, usually associated with vegetation type Elevation could indirectly affect temperature and precipitation between lowlands and uplands	[20, 63–65]
Distance to the Arctic coast	May influence dietary exposure to marine biota, which can have greater Hg concentrations Higher Hg concentrations have been observed in Arctic terrestrial mammals with marine influence in their diet	[14, 15]
Soil Organic Carbon	Organic matter in wet sedge tundra has been identified as a potential site for MeHg production during the spring melt Soil organic matter content is one of the major controls of soil Hg mobilization	[8, 20, 21]
Soil pH	Low pH caused by organic acids in wetlands can inhibit demethylation	[66]

Raster datasets of the landscape and climate factors in Table 1 were acquired or calculated from open data sources, with the exception of Hg deposition (see **Text S1 in** S1 File for a detailed description of these datasets). Spatial variation of net atmospheric Hg deposition and wet Hg deposition (μg/m^2^) in Canada were estimated for the year 2015 with the Global Environmental Multi-scale, Modelling Air quality and Chemistry model (GEM-MACH-Hg) [67]. Modelled net Hg deposition is the balance between gross wet and dry deposition and re-emission to air, while wet deposition reflects gross atmospheric deposition in precipitation. Mercury deposition raster files had a spatial resolution of 0.03 x 0.02 degrees (longitude, latitude) which is approximately 1650 x 2260 m raster cell size. The remaining climate and landscape datasets are described in **Text S1 and Table S1 in** S1 File, as well as the spatial resolution, dates, and source of acquisition. The distance to the Arctic coast (DistCoast) was estimated using vector line features representing the Arctic coastline (**Text S1 in** S1 File). The Euclidean distance from the coastline to the wolverine collection location was calculated to evaluate the Arctic marine influence on food sources and, hence Hg concentrations. Re-projecting, sub-setting, summarizing and pre-processing steps of raster and vector datasets were performed with R [68] using the packages ‘sf’, ‘raster’, ‘exactextractr’ and ‘rgee’ [69–72]. The Tabulate Area tool from the Spatial Analyst in ArcGIS Pro 2.8.2 [73] was used to summarize the land cover classes for each wolverine.

### Data analysis

Raw data generated in this study are provided in S2 and S3 Files.

#### δD as a tracer of wolverine movement

The relationship between hydrogen stable isotope ratios in hair (δD_hair_) of wolverine and precipitation (δD_precip_) was examined to help determine the appropriate spatial scale for the analysis of landscape and climate influences on wolverine THg. Specifically, δD_precip_ was averaged over circular buffer areas of varying sizes (radii) surrounding the wolverine collection site, to identify a scale that optimized the correlation between δD_precip_ and δD_hair_. Isotopic signals from drinking water and prey consumption are integrated during the time of hair growth but there is limited information on hair growth and turnover rate for wolverine [31, 32, 38]. Some have reported a single annual moult in wolverine from late spring to early summer or autumn [74], while another source reports two moulting periods, early spring and autumn [75]. Other members of the Mustelidae have been shown to moult twice a year (spring and autumn), while other species probably undergo only a spring moult [76–78]. At high latitudes, during the shortening days of autumn, the old coats are replaced within days while in more temperate climates the moulting process may take place over a month or six weeks [76]. Similarly, if spring conditions are colder or later than average, the shedding of the old fur can be delayed. We expect wolverine to have a single late spring moult and the guard hairs begin growing in late spring and continue through the fall similar to other temperate large-bodied carnivores [79]. Therefore, two different raster models of δD_precip_ (isoscapes) were tested: mean annual (δD_ma-precip_) and amount-weighted growing season (δD_gs-precip_), to assess if either of these showed a stronger association with the δD_hair_. Growing season refers to months with average temperatures > 0°C [80].

The global δD_ma-precip_ and δD_gs-precip_ isoscapes were obtained from the regionalized cluster-based water isotope prediction (RCWIP) modelling [80, 81] based on the Global Network for Isotopes in Precipitation (GNIP) data (www.iaea.org/water). The grids had a 10 arcmin spatial resolution (~ 9.2 km at 60°N latitude) and were reprojected to North America Albers equal-area conic projection because some of the wolverines were adjacent to the Alaska border. Circular buffers with varying radii around the wolverine collection location were used to summarize pixel values from the δD_ma-precip_ and δD_gs-precip_ isoscapes. The buffer radii tested were 6, 10, 20, 30, 50, 100, 150, 200, 300, 400, 500 km to examine the influence of scale.

Spearman correlation (ρ) was calculated to evaluate the relationship between δD_hair_ and δD_ma-precip_ and δD_gs-precip_ at collection location and mean pixel values for each buffer size. Preliminary results using the δD_hair_ suggested that δD isoscapes may provide only coarse information on habitat use due to low discrimination power associated with small δD gradients of precipitation at high latitudes [82]. Those results also suggested other factors were driving the δD_hair_ signatures in wolverine. Hence, elevation in meters, precipitation accumulation (prcp) in mm and maximum and minimum temperature in °C (Tmax and Tmin) were collected for the collection location and around the buffers sizes to account for other wolverine habitat characteristics. Monthly precipitation and temperature data were averaged over the harvest season for each wolverine. See **Table S1 in** S1 File for datasets used.

Multiple linear regressions (MLR) were performed to identify environmental influences on wolverine δD_hair_. These variables were δD_ma-precip_, DistCoast, minimum elevation, maximum elevation, mean elevation, range elevation, mean prcp, mean Tmax, mean Tmin, latitude and longitude. In addition, biome type (tundra, taiga, boreal and western cordillera; Fig 1), sex and age-class were also included in the initial models to account for those potential intrinsic effects. Stepwise backward variable selection was applied in R. 4.0.2. [68] following recommended best practices [83]. Matrix correlations were conducted to identify collinearity between variable pairs. The number of potential variables was reduced for variable pairs with high collinearity, defined as a Spearman ρ > 0.9. We acknowledge that other thresholds exist to identify substantial correlation among explanatory variables. The collinear explanatory variables generally were different measures of the same type of variable (e.g., minimum versus maximum temperature). All remaining variables were entered into the initial model and the parameters with a p-value > 0.05 were eliminated. After removing the least significant term, all variables with variance inflation factor (VIF) > 10 were dropped, combined with a forward stepwise approach to verify that the most important variables were included. Residuals were checked for linear regression assumptions and details are provided as Supplemental Information (**Text S2 in** S1 File). The relative contribution of the variables from the final model were calculated using the R package ‘relaimpo’ [84]. This analysis produces an estimate of the independent R^2^ for each retained variable. The independent R^2^ values sum to the adjusted R^2^ of the model, providing a good indication of the independent contribution of each variable to total explained variance in the dependent variable.

#### Environmental drivers of THg in wolverine muscle

Using the environmental and climate factors (Table 1), 18 potential explanatory variables for THg in wolverine muscle were calculated for the selected buffer area (based on the δD_hair_ analysis described above) around each collection location. The potential environmental variables were: mean net and wet Hg deposition, mean prcp, mean Tmax, mean Tmin, mean elevation, DistCoast, mean soil organic carbon (SOC), mean subsoil pH at 60 cm depth (spH60), mean topsoil pH at 10 cm depth (spH10) and percentage of land cover types (forest %, shrubland %, grassland %, barren land %, wetland %, water %, wet area %, and snow-ice %). A description of each potential model parameter calculation is provided in **Table S2 in** S1 File. Covariates for biome type, sex and age class were also included. Measurements of δ^15^N and δ^13^C in wolverine muscle were also added as potential predictor variables to assess the influence of dietary sources on spatial patterns of wolverine THg concentrations. Spearman correlations were calculated between wolverine THg concentrations and each explanatory variable to examine co-variance. MLRs were performed for wolverine THg (natural-log transformed) using backward stepwise selection as previously described.

## Results

### δD in wolverine hair

The δD_hair_ values for wolverine varied from -146 to -91‰ (Table 2). There was no difference between male and female δD_hair_ values (Mann-Whitney-Wilcoxon U = 768.5, p = 0.93; **Fig S1B in** S1 File). Age class information was available for 68 (85%) of 80 wolverines examined for δD, (26 sub-adults and 42 adults; Table 2) and the δD_hair_ values were significantly different between age classes (Mann-Whitney-Wilcoxon U = 374, p = 0.03; **Fig S1A in** S1 File); with lower values in sub-adults than in adults.

**Table 2:** Stable isotope ratios of hydrogen (δD_h_) measured in wolverine (*Gulo gulo*) hair across boreal and Arctic biomes in western Canada, by sex and age.

δD_hair_ (‰)	Female			Male		
	Adult	Sub-adult	Unknown	Adult	Sub-adult	Unknown
	n = 16	n = 8	n = 7	n = 26	n = 18	n = 5
Mean	-122	-125	-106	-119	-125	-104
(SD)	(8)	(8)	(10)	(12)	(11)	(2)
Range	-136,	-136,	-128,	-146,	-141,	-107,
	-107	-112	-98	-99	-91	-102

A Kruskal-Wallis test showed significant differences in δD_hair_ values of wolverines between biome types (H_3_) = 35.62, p < 0.001; **Fig S1C in** S1 File). Thus, Wilcoxon’s pairwise rank-sum tests were carried out to compare all pairs of groups. There was strong evidence (p < 0.01, using Hochberg’s post hoc adjustment method) of a difference between δD_hair_ in boreal cordillera and in tundra and western cordillera. Western cordillera δD_hair_ values showed a significant difference from those in tundra and taiga. The differences between taiga and boreal cordillera, and between taiga and tundra were not significant.

**Fig 2:**
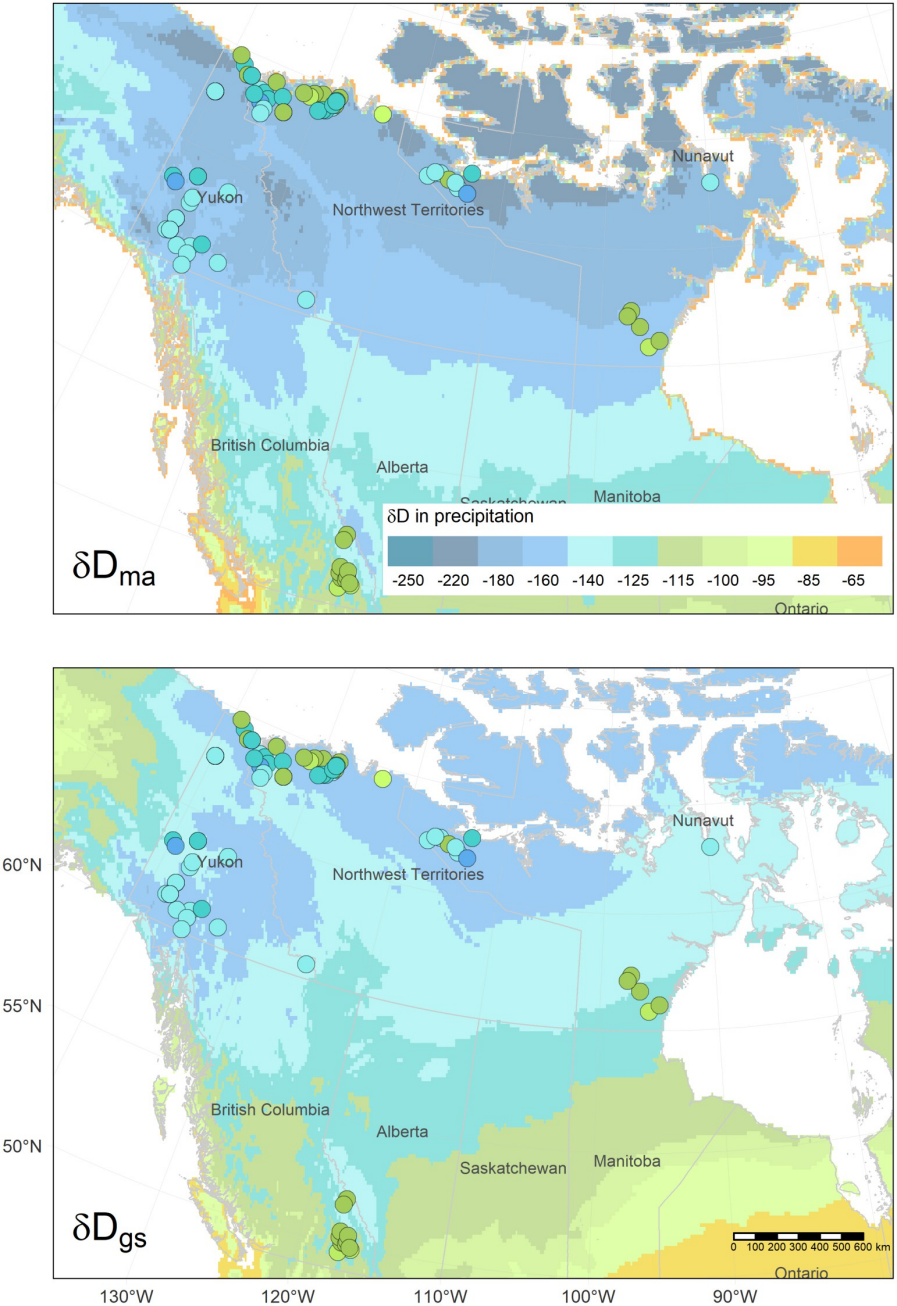
Hydrogen isotope isoscapes of mean annual (δD_ma-precip_) and weighted growing season (δD_gs-precip_) precipitation from the RCWIP model [81]. Legend is δD in ‰. Circles represent δD of wolverine (*Gulo gulo*) hair; values were scaled to the same legend for δD of precipitation.

**Fig 3:**
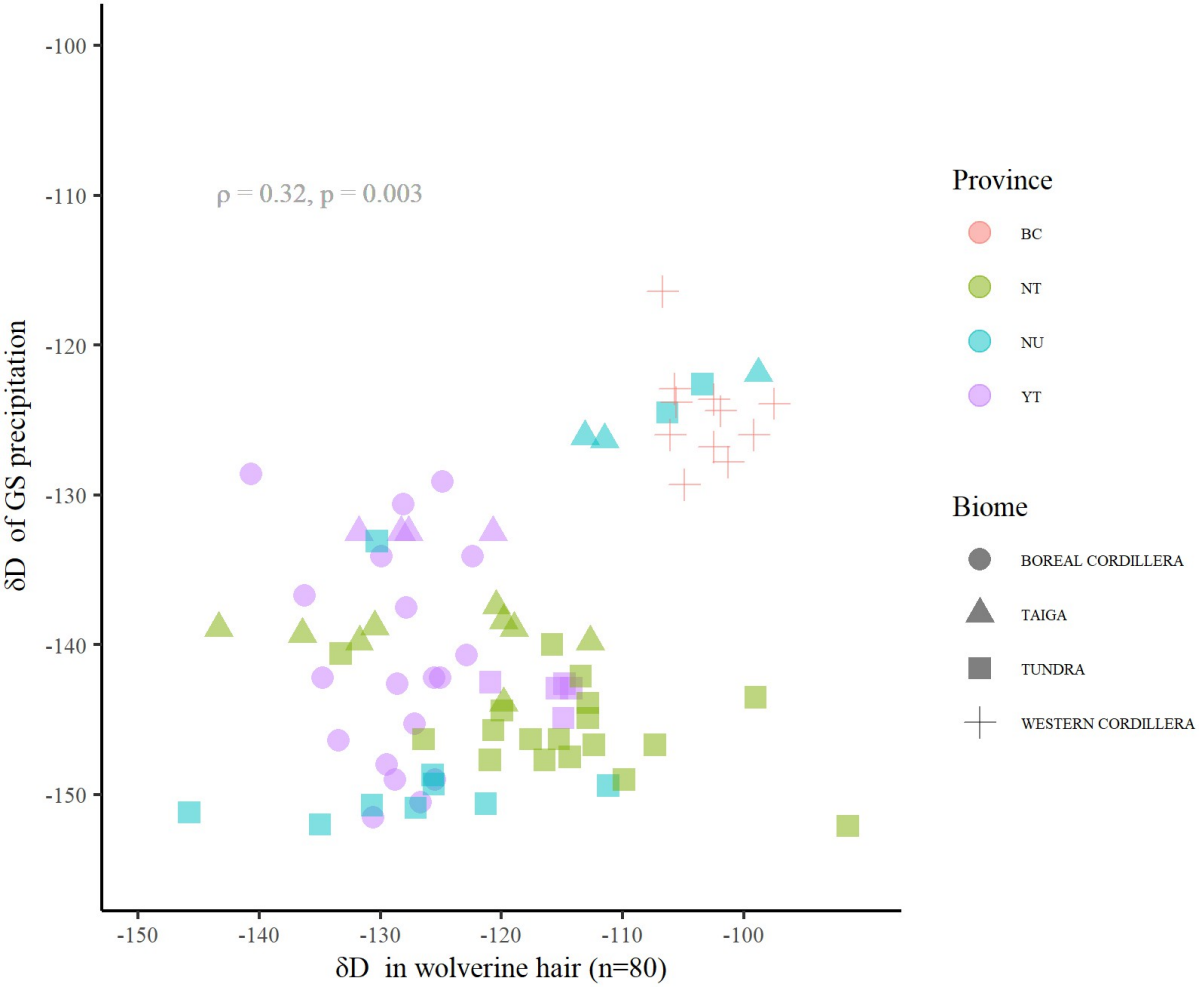
Hydrogen isotope ratios of wolverine (*Gulo gulo*) hair (δD_hair_) and growing season precipitation (δD_gs-precip_) at collection locations across boreal and Arctic biomes in western Canada.

**Fig 4:**
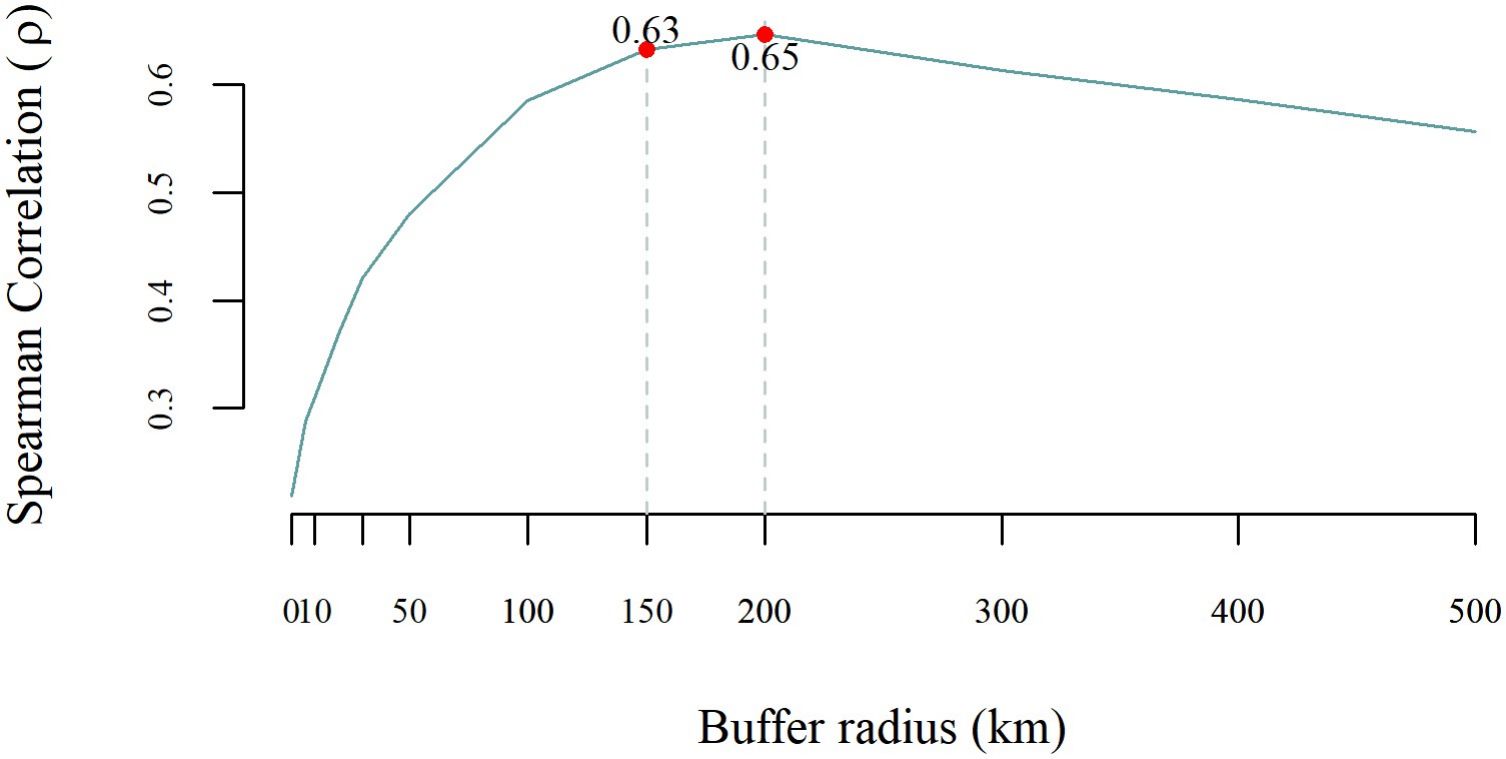
Spearman correlation coefficients (ρ) between hydrogen isotope ratios of wolverine (*Gulo gulo*) hair (δD_hair_) and mean annual precipitation (δD_ma-precip_) around varying buffer radii from the collection location.

The δD_hair_ of wolverine was related to the two δD_precip_ isoscapes (δD_ma-precip_ and δD_gs-precip_) (Fig 2), although the strength of the associations varied between isoscapes and buffer sizes (Table 3). At the point scale (collection location), δD_hair_ and δD_gs-precip_ precipitation were significantly correlated (ρ = 0.32, p < 0.01; Fig 3). The correlation strength was similar for δD_gs-precip_ measurements at buffer sizes from 6 km to 200 km. For example, at the 150 km buffer size the correlation coefficient was similar to that for the collection location. On the other hand, the correlation between δD_hair_ and δD_ma-precip_ varied at different scales, at the collection location the association was not significant but became significant and stronger when summarizing values around larger buffers from the wolverine collection location (Fig 4). The 150 and 200 km buffer radius showed the strongest correlations between δD_hair_ and δD_ma-precip_ (ρ = 0.63 and 0.65 respectively, p ≤ 0.001) (Table 3, Fig 4). The δD_ma-precip_ isoscape was selected for further analysis due to the stronger correlation with δD_hair_ at increasing buffer radii. The difference between δD_hair_ of wolverine and collection location δD_ma-precip_ was ~ 43 ± 15 ‰, and at the 150 km buffer scale the difference was ~23 ± 18 ‰.

**Table 3:** Spearman coefficients (ρ) for correlations of the δD_hair_ of wolverine (*Gulo gulo*; n = 80) with δD of precipitation, and climate and physiographic variables.

Variable	Collection location		150 km buffer	
	ρ	p-value	ρ	p-value
δD_gs-precip_	**0.32**	**0.003**	**0.32**	**0.004**
δD_ma-precip_	0.22	0.052	**0.63**	**≤ 0.001**
prcp	0.09	0.427	0.01	0.943
Tmax	**-0.24**	**0.031**	0.03	0.789
Tmin	-0.17	0.137	0.15	0.179
Long	**0.46**	**≤ 0.001**		
Lat	-0.03	0.79		
Elevation	-0.09	0.442		
DistCoast	-0.05	0.652		
Max Elevation			-0.12	0.294
Min Elevation			0.06	0.568
Mean Elevation			-0.12	0.277
Range Elevation			-0.11	0.322

Correlation matrices of the explanatory variables indicated high multi-collinearity among some variables (**Figs S2 and S3 in** S1 File). At the collection location scale, there was strong covariance between Tmax and Tmin (ρ = 0.94), and between latitude and DistCoast (ρ = -0.91; **Fig S2 in** S1 File). Hence, for the MLR global model at collection location, Tmin, and latitude were removed. Around the 150 km buffer there was a strong correlation between maximum elevation and range elevation (ρ = 0.99; **Fig S3 in** S1 File), between Tmax and Tmin (ρ = 0.92), and between latitude and prcp (ρ = 0.95). Further, for the MLR at the 150 km buffer, range elevation, Tmax, and prcp were removed from the global model.

Multiple linear regression models, performed at two scales (the collection location and 150 km buffer), indicated that climate and physiographic variables explained variation in δD_hair_ in addition to δD_ma-precip_. The best fit model to explain δD_hair_ in wolverines based on collection location measurements included two variables: δD_ma-precip_, and Tmax. The model accounted for approximately 55% of the variation in δD_hair_ of wolverine. Overall, δD_ma-precip_ explained slightly more of the variance in δD_hair_ (~ 28%, based on independent R^2^; Table 4). The positive direction of the beta coefficient shows that δD_hair_ increased with increasing δD_ma-precip_. Maximum temperature contributed ~ 26% of the δD_hair_ variation in wolverines (Table 4). The negative beta coefficient for this variable indicates decreasing δD_hair_ in wolverines with increasing values of Tmax.

**Table 4:** Modelled explanatory variables of δD_hair_ of wolverine (*Gulo gulo*) at the collection location and the 150 km buffer scales.

		Collection location							150 km buffer					
Variable	Beta	Estimates	SE^1^	t-val	p-val	VIF^1^	Independent R^2^	Beta	Estimates	SE^1^	t-val	p-val	VIF^1^	Independent R^2^
(Intercept)	0.000	-13.8	12.7	-1.09	0.3			0.000	6.05	13.1	0.46	0.6		
δD_ma-precip_	0.697	0.573	0.073	7.88	**<0.001**	1.2	0.28	0.503	0.267	0.040	6.68	**<0.001**	1.1	0.33
Tmax	-0.681	-1.46	0.190	-7.70	**<0.001**	1.2	0.26							
Lat								-0.707	-1.24	0.188	-6.60	**<0.001**	2.2	0.23
Max Elev								-0.481	-0.005	0.001	-4.57	**<0.001**	2.1	0.07
R^2^		0.55							0.63					
Adjusted R^2^		0.53							0.61					
Residual SE		7.48							6.99					
F-statistic		42.6							39.6					
p-val.		<0.001							<0.001					
n		74							74					

^1^SE = Standard Error, VIF = Variance Inflation Factor

At the 150 km buffer scale, the best fit model explained 63% of the variation in δD_hair_ of wolverine and included three variables: δD_ma-precip_, latitude and maximum elevation (Table 4). Similar to the other model, δD_ma-precip_ was the most important variable as well (independent R^2^ = 0.33), with a positive effect (beta coefficient = 0.50) on the variation in δD_hair_. Including latitude (independent R^2^ = 0.23) and maximum elevation (independent R^2^ = 0.07) to the 150 km buffer model provided a combined 30% increase in explained variability in δD_hair_. The negative beta coefficients for maximum elevation and latitude point to decreasing δD_hair_ in wolverines with increasing values of these explanatory variables.

### THg in wolverine muscle

**Fig 5:**
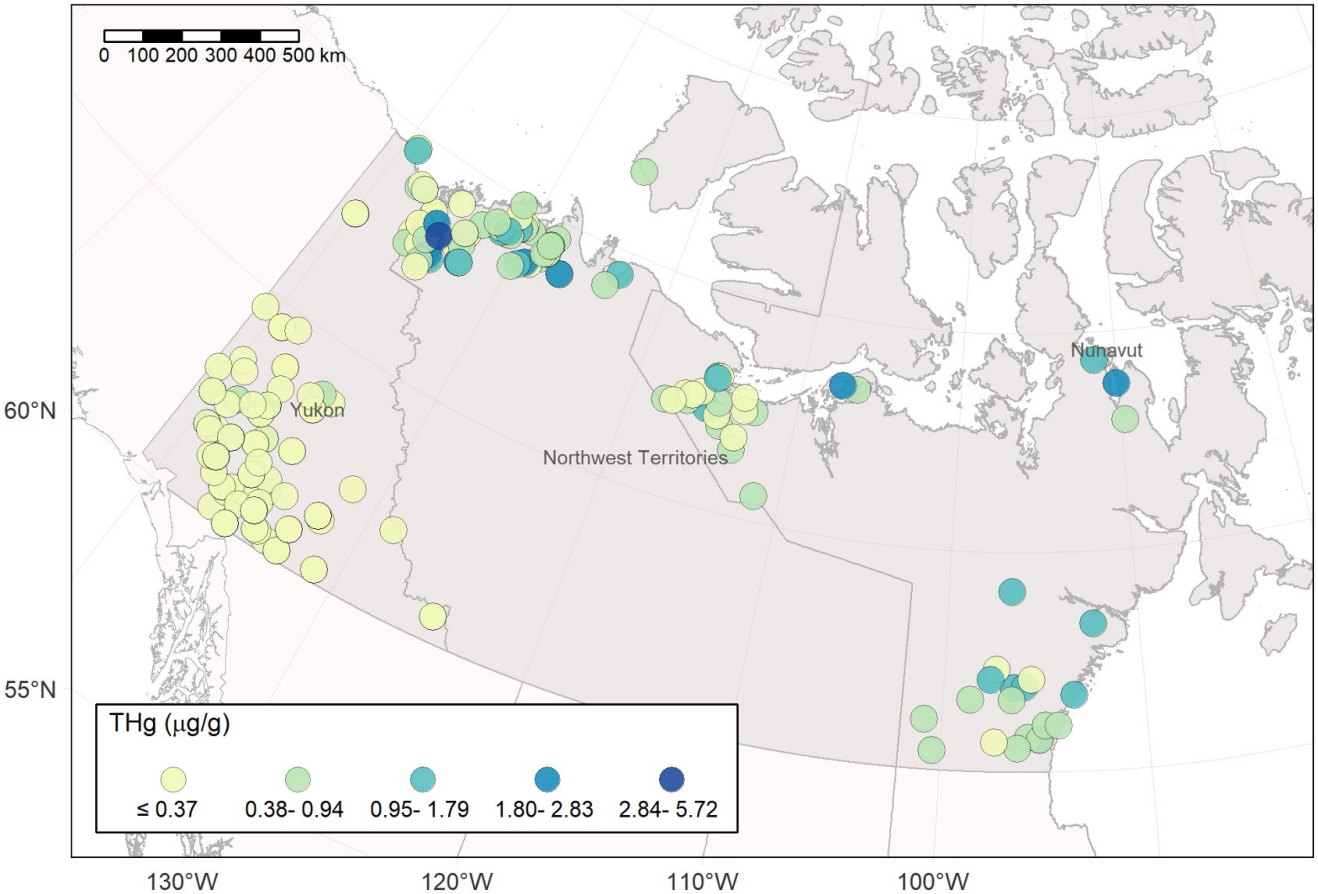
Total Hg concentration (μg/g dw) in wolverine (*Gulo gulo*) muscle (n = 419) across boreal and Arctic biomes in western Canada.

Total Hg concentration in wolverine muscle varied over two orders of magnitude within the study area, ranging from 0.01 to 5.72 μg/g dw (Fig 5, **Table S3 in** S1 File) and also varied up to three-fold between territories. The highest average concentration was measured in the NT (mean = 0.94 ± 1 μg/g dw, median = 0.62) while wolverines in YT had the lowest THg concentrations in muscle (mean = 0.22 ± 0.26 μg/g dw, median = 0.13). A Kruskal-Wallis test (H_2_ = 123.15, p < 0.001) followed by a Wilcoxon’s pairwise rank-sum test (p < 0.01, using Hochberg’s post hoc adjustment method) indicated significantly lower THg in YT compared with the other two territories (**Fig S4C in** S1 File). The difference in THg between NU and the NT were not significant. When grouped by biome, wolverines in the boreal cordillera had significantly lower THg concentrations than those in taiga and tundra (H_2_ = 113.95, p < 0.001; Wilcoxon’s p ≤ 0.01, Hochberg’s post hoc).

Total Hg values were significantly different between age classes (U = 17691, p = 0.04; **Fig S4A in** S1 File), with sub-adults having on average lower THg concentrations than adults. Age-class was available for most wolverines (406 of 419, 96%): 152 were sub-adults and 254 were adults (Table S3 in S1 File). There was no difference in THg concentration between males and females (U = 17850, p = 0.08; **Fig S4B in** S1 File).

**Fig 6:**
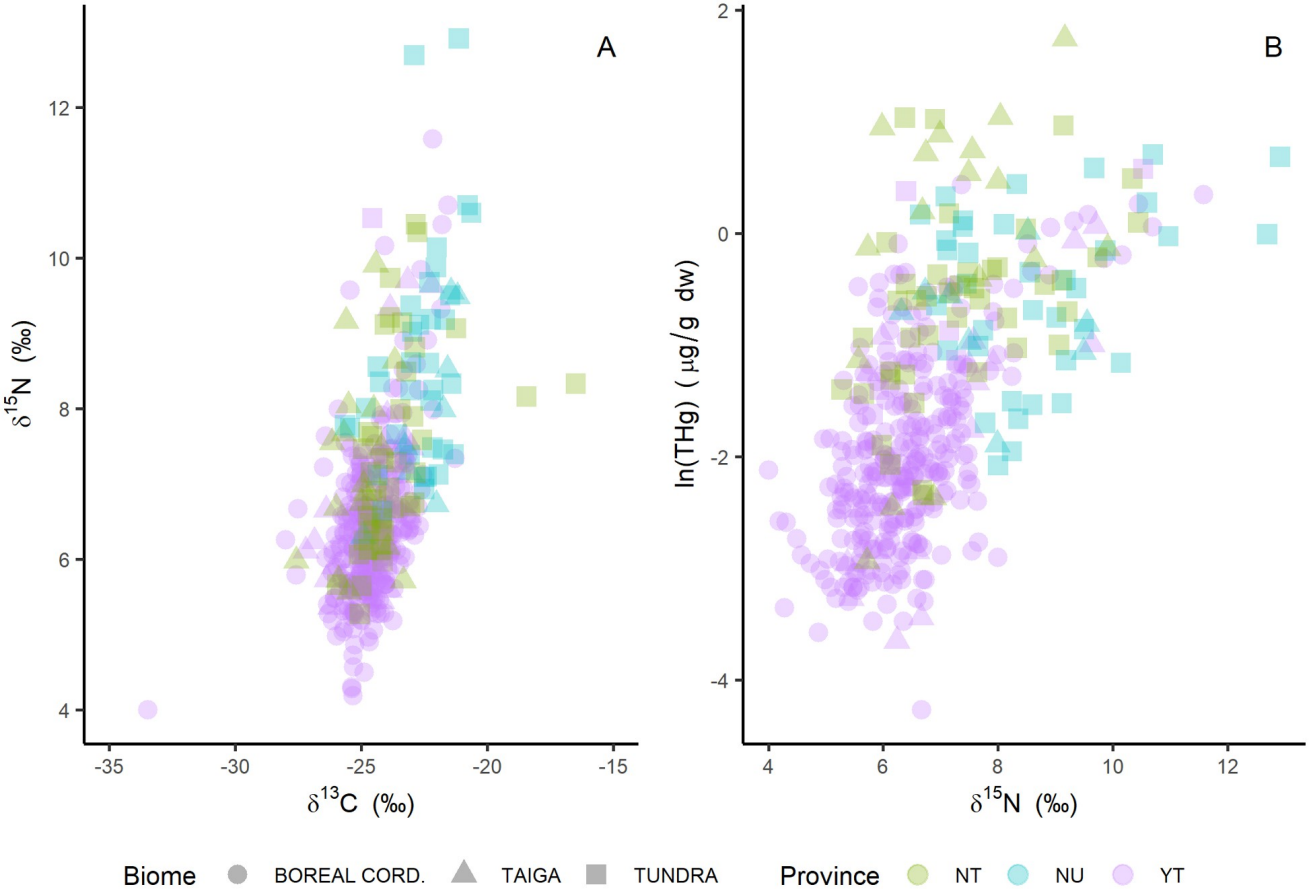
Diet variation and influence on wolverine THg using carbon and nitrogen stable isotopes. A: Nitrogen and carbon stable isotope composition (δ^15^N and δ^13^C) of wolverine (*Gulo gulo*) muscle across boreal and Arctic ecosystems in Canada. B: ln(THg) concentration in wolverine muscle in relation to δ^15^N. One outlier was removed from panel A (δ^13^C = -8.04‰).

The δ^15^N and δ^13^C of wolverine muscle indicated differences in diet between biomes. The δ^15^N and δ^13^C values ranged from 3.99 to 12.91 ‰ (mean = 6.79‰) and from -33.46 to -8.04 ‰ (mean = -24.29‰), respectively (Fig 6A). The δ^15^N of wolverine muscle was significantly higher in the tundra in comparison with the taiga and boreal cordillera wolverines (H_2_ = 79.07, p < 0.001; Wilcoxon’s pairwise rank-sum tests p < 0.001, using Hochberg’s post hoc). The δ^15^N values in wolverines from boreal cordillera were significantly lower than in the other two biomes. Carbon stable isotope ratios in wolverine muscle were significantly higher in the tundra biome compared to the taiga and boreal cordillera biomes (H_2_ = 54.03, p < 0.001; Wilcoxon’s p ≤ 0.001, using Hochberg’s post hoc). There was no significant difference in δ^13^C values between boreal cordillera and taiga wolverines (p = 0.29). Wolverine THg concentrations were strongly correlated with δ^15^N (Fig 6B, Table 5).

**Table 5:** Spearman coefficients (ρ) for correlations of climate, landscape, and diet variables with THg concentration in wolverine (*Gulo gulo*) muscle (n = 419).

Variable	Collection location		150 km buffer	
	ρ	p-value	ρ	p-value
Hg-net deposition	0.02	0.732	**0.12**	**0.017**
Hg-wet deposition	**0.37**	**≤ 0.001**	**0.49**	**≤ 0.001**
prcp	***-0*.*35***	**≤ 0.001**	***-0*.*41***	**≤ 0.001**
Tmax	**-0.48**	**≤ 0.001**	**-0.52**	**≤ 0.001**
Tmin	**-0.46**	**≤ 0.001**	**-0.5**	**≤ 0.001**
Elevation	**-0.44**	**≤ 0.001**	**-0.45**	**≤ 0.001**
SOC	0.08	0.092	**0.19**	**≤ 0.001**
spH10	-0.07	0.128	**-0.15**	**0.003**
spH60	-0.07	0.166	**-0.17**	**≤ 0.001**
δ^15^N	**0.6**	**≤ 0.001**		
δ^13^C	**0.41**	**≤ 0.001**		
DistCoast	**-0.49**	**≤ 0.001**		
Long	**0.27**	**≤ 0.001**		
Lat	**0.41**	**≤ 0.001**		
forest %			**-0.46**	**≤ 0.001**
shrubland %			**0.23**	**≤ 0.001**
grassland %			**0.4**	**≤ 0.001**
barren land %			**-0.24**	**≤ 0.001**
wetland %			**0.47**	**≤ 0.001**
water %			**0.43**	**≤ 0.001**
snow-ice %			**-0.38**	**≤ 0.001**
wet area %			**0.48**	**≤ 0.001**

Wolverine THg showed a moderate positive correlation with wet Hg deposition at the collection location (ρ = 0.37, p < 0.001) and within the 150 km buffer (ρ = 0.49, p < 0.001). In contrast, net Hg deposition was not significantly correlated to THg when measured at the wolverine collection location. At the 150 km buffer scale, the correlation between these two variables was significant but the effect was small (ρ = 0.12, p = 0.017). In addition, THg concentrations in wolverine muscle were correlated with many landscape and climate variables (Table 5). Wolverine THg was significantly positive correlated with latitude and longitude. Distance to the Arctic coast, Tmax, Tmin, elevation and prcp were also significant but negatively correlated with THg. The soil variables (SOC, spH10, spH60) were not significantly correlated with THg at the collection location but were weakly correlated with THg at the 150 km buffer scale. All the land cover variables were moderately correlated with THg. The association was positive between THg and shrubland %, grassland %, wetland %, water %, and wet area %. Barren land % and snow-ice % had a negative correlation with THg in wolverine.

**Fig 7:**
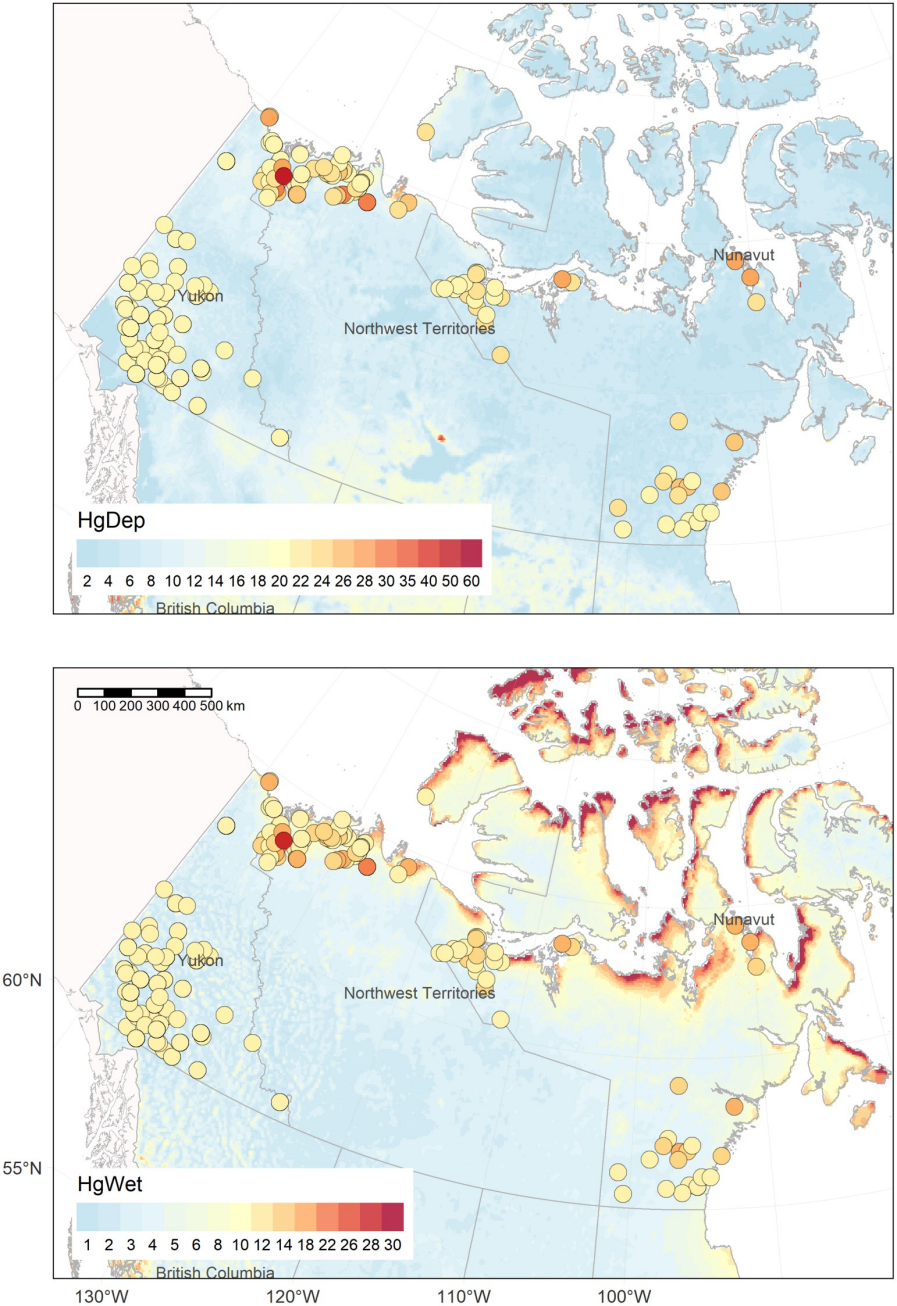
Annual net Hg deposition (HgDep) and wet Hg deposition (HgWet) for 2015 in Canada (GEM-MACH-Hg model). Legend is in μg/m^2^. Circles represent muscle THg concentrations of wolverines (μg/g). Wolverine (*Gulo gulo*) THg values were scaled to the same legend for Hg deposition (for HgDep, THg was offset by 3.5X + 20 and for HgWet by 3X + 10).

Wolverines closer to coastal areas in the NT and NU had higher THg concentrations than wolverines in inland areas (Figs 5 and 7). Distance to the Arctic coast showed collinearity with other explanatory variables, such as wet Hg deposition, temperature, precipitation, elevation and δ^15^N (**Fig S5 in** S1 File). Modelling estimates of net and wet Hg deposition are shown in Fig 7 while **Fig S7 in** S1 File shows the climate and elevation models. Multiple environmental variables and diet may contribute to the coastal habitat influence on wolverine THg. For example, northern coastal areas receive greater atmospheric wet deposition of Hg compared with inland areas (Fig 7).

There was strong covariance among explanatory variables at the two spatial scales examined. At the collection location scale, there was strong covariance between Tmax and Tmin, between spH10 and spH60, and between latitude and DistCoast (**Fig S5 in** S1 File). To reduce the set of explanatory variables in the MLR models applied at the collection location scale, Tmin, spH60, and latitude were removed. Seventeen outliers were removed from the final model. At the 150 km buffer scale, there was strong collinearity between elevation and snow-ice %, between spH10 and spH60 and between Tmax and Tmin (**Fig S6 in** S1 File). Accordingly, elevation, Tmin, and spH60 were removed for the MLR modelling at the 150 km buffer scale. Thirteen outliers were removed from the final model at this scale.

The best fit models for ln(THg) at the two spatial scales examined indicated diet and landscape variables influenced THg bioaccumulation in wolverine muscle. At the collection location scale, ln(THg) was influenced by three variables: SOC, DistCoast and δ^15^N, explaining approximately 45% of the variation (Table 6). The δ^15^N was the most important variable for explaining ln(THg) variation in wolverines at this scale (independent R^2^ = 0.23) and the association was positive. At the collection location, the beta coefficient for DistCoast (independent R^2^ = 0.20) was negative, indicating a moderate inverse association with ln(THg) in wolverines. Soil organic carbon contributed modestly but significantly and positively to ln(THg) variation at the collection location scale (independent R^2^ = 0.02).

**Table 6:** Factors that influence ln(THg) of wolverine (*Gulo gulo*) at collection location and the 150 km buffer.

		Collection location							150 km buffer					
Variable	Beta	Estimates	SE^1^	t-val	p-val	VIF^1^	Independent R^2^	Beta	Estimates	SE^1^	t-val	p-val	VIF^1^	Independent R^2^
(Intercept)	0.000	-3.97	0.333	-11.9	**<0.001**			0.000	-5.49	0.269	-20.4	**<0.001**		
SOC	0.142	0.004	0.001	3.80	**<0.001**	1.0	0.02	0.206	0.008	0.002	5.24	**<0.001**	1.2	0.07
DistCoast	-0.351	0.000	0.000	-8.29	**<0.001**	1.3	0.20							
δ^15^N	0.419	0.389	0.040	9.84	**<0.001**	1.3	0.23	0.449	0.384	0.034	11.4	**<0.001**	1.3	0.24
Snow-ice %								0.134	0.022	0.007	3.33	**<0.001**	1.3	0.02
Wet area %								0.337	0.050	0.007	7.59	**<0.001**	1.6	0.18
R^2^		0.45							0.50					
Adjusted R^2^		0.44							0.50					
Residual SE		0.775							0.734					
F-statistic		107							101					
p-val.		<0.001							<0.001					
n		402							406					

^1^SE = Standard Error, VIF = Variance Inflation Factor

The best fit model for ln(THg) in wolverine muscle at the 150 km buffer scale explained 50% of the variation and included four variables: SOC, δ^15^N, snow-ice % and wet area % (Table 6). Nitrogen isotope composition remained the strongest explanatory variable (independent R^2^ = 0.24). Percentage of wet area, SOC and percentage of perennial snow-ice showed positive effects (beta coefficients) on ln(THg) of wolverine at this scale. An increasing percentage of these land cover types and SOC may be related to higher ln(THg), especially wet area, which was the second most important variable for the 150 km buffer model (independent R^2^ = 0.18). At the 150 km buffer, distance to the Arctic coast was not selected as a significant explanatory variable.

## Discussion

### Hydrogen isotopes as a tool to constrain movement of Arctic wolverine

A main finding of our study was that δD_hair_ in wolverine exhibited a positive but weak relationship with δD_precip_. This finding differs from strong positive relationships found for keratin tissues of other terrestrial mammals such as bats and felids [85–87], though that is not always the case. In the eastern United States, Britzke et al. [88] found that δD in precipitation explained very little variation in δD_hair_ in two bat species (*Lasiurus borealis* and *Myotis lucifugus*) but the relationship was strong for two other species (*M*. *sodalis* and *M*. *septentrionalis*). Pietsch et al. [89] concluded that feline cannot be placed on δD isoscapes because they did not find any relationship between δD in environmental water and δD_hair_ for puma (*Puma concolor*) and bobcat (*Lynx rufus*) museum samples. In the case of wolverines, the weak to moderate positive correlation of δD_hair_ with δD_ma-precip_ and δD_gs-precip_ indicates that it would not be possible to estimate the origin of wolverine if the collection location was not already available. Given that wolverines are territorial [90] and considering their typical home range size (from 73 to 3,513 km^2^; [91]), it is unlikely that the animals dispersed across large latitudinal gradients, which are often examined for migratory wildlife and needed for clear isotopic discrimination [32, 38, 92, 93]. Nevertheless, wolverine δD_hair_ was helpful to identify a suitable spatial scale (scale of environmental influence) for the THg analysis.

Our application of δD as a means of providing insight on a suitable spatial scale for studying contaminant exposure is a novel approach that could be useful to expand H stable isotope applications for terrestrial wildlife. There are advantages and limitations associated with this application for wolverines. First, studies of wolverine movement and habitat derived from direct tracking and modeling techniques often report varying home ranges, dispersion distances and habitat use among individuals across North America. The use of δD_hair_ allowed for an estimation of a general area of habitat influence for wolverines that was independent of the statistical modelling for Hg, and therefore provided additional validation. Incorporating different buffers sizes per sex or age-class may have relevance, due to distinct home ranges sizes and dispersal movement’s habits, although these biological variables were not significant in explaining the H stable isotope models. Additionally, wolverine home range and habitat use may vary among biome (e.g., tundra vs boreal) but this was not considered either. Nevertheless, the 150 km radius scale model generated more information and reduced variance of the Hg model compared to using the collection location.

A factor that could explain the weak relationship between δD_precip_ isoscapes and δD_hair_ in wolverine is the intrinsic discrimination between precipitation and hair [38]. Poorly characterized species-specific moulting pattern (e.g., tissue growth and isotopic integration periods) can be another source of variation that was not part of this study but is important for interpreting isotopic data [87, 94]. Isotope fractionation and routing during metabolism and tissue formation is likely to control the δD_hair_ composition of wolverine. Metabolic and physiological processes associated with the transfer of H through the body water of an animal affect the incorporation of H in hair tissue [38, 95]. Physiological effects (e.g., synthesis of amino acids and keratins) and greater metabolism in strict carnivores have been hypothesized to explain weaker relationships between δD_hair_ and δD_precip_ in comparison to terrestrial omnivores and herbivores [86, 89, 95, 96]. Laboratory controlled-feeding experiments have found that assimilation and discrimination of δD vary between tissues and among the sources of H (water or dietary) [96]. Such experiments in carnivores are currently lacking. As primary carnivores, the relationship between δD_hair_ of wolverines and environmental water can be complicated by unknown metabolic and physiological processes in the transfer of H.

Source pools of H can be another factor contributing, to different extents, to the δD composition in wolverine hair. Mammals have three main sources of H in body water (thus hair) that is available for H exchange. Hydrogen can be obtained from drinking water, water incorporated from food, and metabolized food, with the relative importance of sources varying among species [86, 97]. Drinking water supplies >55% of the H atoms of human body water [95, 98], contributing to ~27% of the H in human hair keratin; while the remaining fraction of H originates from blood water and metabolism of dietary foods [97]. Consequently, as long as individuals consume a local diet, the relationship between δD_hair_ in human and δD in water facilitates ecological applications of this tool. Strict carnivores, on the other hand acquire most of their body water intake from food, rather than drinking water [86, 99]. For instance, polar bears do not drink fresh water and produce body water by consuming a high-fat diet [99]. In domestic cats, Koehler & Hobson [86] found a weaker relationship of δD_hair_ with δD_precip_ than for domestic dogs. Cats, as strict carnivores obtain most of their water requirements from prey consumption and consume little water to facilitate the excretion of metabolic end-products while dogs as omnivores consume more drinking water [86, 89]. Variation in δD_hair_ of wolverines might also be attributed to varying inputs of diet sources (e.g., terrestrial, freshwater or marine). For example, a high animal protein consumption and a marine-based diet may have influenced the anomalous δD values in Inuit hair samples compared with other global human populations [95]. Similarly, marine protein and fat intake may have complicated the δD_hair_ signatures in wolverine. Understanding how diet influences assimilation and discrimination of δD in terrestrial mammals is an important step toward applying it as a tracer for wildlife [96].

Climate and physiographic factors, specifically latitude, elevation and temperature, explained some of the variation in δD_hair_ of wolverine. The influence of latitude suggests there is a north-south gradient in δD_hair_ in wolverines that was not captured by the δD_ma-precip_. For bats, latitude explained more variability in δD_hair_ than δD_precip_ [88]. A limitation of the current modelling of δD_precip_ isoscapes is the sparse GNIP stations and fragmented data available at high latitudes [80]. Hence, there are large uncertainties in the spatial interpolation of δD_precip_ in this region. The influence of elevation has been reported elsewhere for δD in the feathers of birds [100] and δD in human body water [98]. Since wolverines forage at a variety of elevations and topographic ruggedness between seasons and habitats types [40, 45, 101–104], this variable may reflect individual habitat use that was not captured by the other variables. The influence of temperature in δD_hair_ is unclear but it is probable that affects the geographic distribution of δD_precip_ (e.g., water condensation) [105, 106] or metabolic processes in wolverines (e.g., excretion and evaporation of δD) [98, 107]. Therefore, the accurate spatial prediction of δD in body water (and hair) in mammalians may require the incorporation of key sources of hydrogen and the influence of climate.

### Wolverine muscle THg concentrations in the context of the Arctic

Average THg concentrations in wolverine muscle were higher than those reported in herbivores and lower than those in the muscle of Arctic marine carnivores (**Table S4 in** S1 File). However, mean (0.37) and median (0.17) values of THg (μg/g dw) in wolverine muscle from this study were similar to or higher than the average concentrations reported in muscle from other terrestrial large predators (**Table S4 in** S1 File). Total Hg in red fox (*Vulpes vulpes*) muscle averaged from 0.05 to ~0.08 μg/g dw [108–110], while mean and median values in gray wolf muscle were ~0.33 and ~0.05 μg/g dw [15, 111]. For both canine species, greater average THg levels were documented in animals with access to coastal areas and presumably to marine food webs. The highest muscle THg concentration recorded for wolverine (5.72 μg/g dw) exceeded the highest values reported in the muscle of red fox (3.6 μg/g dw) and gray wolf (~2.2 μg/g dw) that had coastal influences [15, 109]. Nine wolverines exhibited THg concentrations ≥2 μg/g dw; all were in tundra biomes from NU and the NT and within ~100 km from the Arctic coast. Total Hg concentrations in wolverine were lower compared to muscle of other North American mustelids (**Table S4 in** S1 File). River otter (*Lontra canadensis*) can have muscle THg concentrations between ~1.28 and ~5.12 μg/g dw [112, 113] while those of mink (*Neogale vison*) were reported between ~2.2 and ~8.5 μg/g dw [113, 114] and for marten (*Martes americana*) were ~1.12 μg/g dw [115]. The higher THg concentrations in otter and mink are likely related to their diet of marine or freshwater fish, whereas marten are terrestrial predators like the wolverine. Average THg concentrations in wolverine muscle are closer to those reported in canine carnivores than in other mustelids and are consistent with those of gray wolf in the Arctic, a terrestrial carnivore that primarily eats large herbivore prey and may opportunistically feed on marine sources, similar to wolverine.

The higher THg concentrations in adult wolverines than in sub-adults is consistent with previous findings from various tissue types in ringed seal (*Pusa hispida*) [34], polar bear [116, 117], arctic fox [14], and other terrestrial mammals [60, 111]. However, the difference between age classes was low (~0.05 μg/g dw) and caution is advised in interpreting this result due to the uneven sampling design (the majority of wolverines were adult in the dataset). Similar to the findings in this study, other Arctic and terrestrial wildlife did not display significant differences in THg concentrations between females and males [13–15, 60, 108, 118]. Note results for other Arctic mammals and mustelids are mixed regarding differences in THg concentrations between sexes and age [34, 112, 116].

The risk of toxicological effects to wolverine from Hg exposure was not assessed using our muscle data because this analysis is commonly conducted on liver or hair for mammalian wildlife [119]. Mercury was measured in brain, liver, and hair for a subset of wolverine from our study, though those data will be reported elsewhere and will include an assessment of toxicological risk. Elsewhere, a small proportion of Arctic fox were reported to have elevated liver Hg concentrations associated with a higher risk of toxicological effects [119], suggesting that an assessment for wolverine with elevated tissue burdens of Hg is warranted.

### Mercury concentrations in wolverine were primarily related to diet

Total Hg in wolverine muscle increased with δ^15^N, which was the dominant explanatory variable at both the collection location and the 150 km buffer scales. These results suggest that THg levels in wolverines reflect food sources and biomagnification processes, widely documented in other Arctic wildlife [11, 34]. Typically, δ^15^N values are more enriched in marine biota relative to terrestrial biota [38, 57]. Following this principle, the δ^15^N values of boreal and taiga wolverines were lower than those in tundra biomes where the latter were more likely to have access to marine diet items. The variation in δ^15^N values of wolverine probably reflect differences in baseline nitrogen isotope signatures of marine, freshwater and terrestrial foods webs but also may reflect differences in trophic position since nitrogen isotope ratios increase by 2.5–5 ‰ per trophic level [36–38].

Although δ^13^C was not an important explanatory variable for our model of THg in wolverine, the significant positive correlation with THg suggested that marine food sources are associated with higher THg levels. Terrestrial and freshwater biomes tend to be more depleted for δ^13^C compared to marine food webs [31, 120] and δ^13^C vs. δ^15^N values are typically correlated in marine systems [57]. For wolverines, the increasing δ^13^C and δ^15^N values (Fig 6) provide evidence of access to marine prey. Similarly, marine diet inputs have been related with higher THg concentrations in other Arctic terrestrial predators [13, 15]. When animals have access to both terrestrial and marine resources δ^13^C values may reflect the relative proportion of marine or terrestrial foods in the diet, while δ^15^N values reflect the trophic position [31, 57].

The large ranges of δ^15^N and δ^13^C values in muscle indicate diet variation in wolverines across our vast study area, though a detailed diet analysis (including isotope mixing models) will be presented elsewhere. Wolverine are an opportunistic, generalist carnivore, with considerable plasticity of diet among and within regions and seasons [55, 91, 102, 121]. Ungulates are often their dominant food source throughout their geographic range, e.g., caribou and moose (*Alces americanus*), especially during the winter [55, 91, 102, 121, 122]. However, in YT, snowshoe hare can be another prominent prey during winter [123]. During summer, medium and small-sized vertebrates have been identified as wolverine primary prey, where available [41, 91, 122, 124]. Mean values of δ^15^N (6.79‰) and δ^13^C (-24.29‰) in wolverine probably reflect a diet of terrestrial herbivores dominated by small terrestrial vertebrates [11, 103, 124]. These results are similar to those from Alaska, which reported average δ^15^N and δ^13^C of 6.50‰ and -22.68‰, respectively, in wolverine muscle [124]. Wolverine use of coastal habitats and feeding habits are not well-documented [46, 125] but our findings suggest marine sources may increase their THg concentrations. The significant relationship between DistCoast and THg also suggested a marine influence on Hg exposure in wolverines. A similar approach found higher THg loads in muscle from coastal vs. inland wolves in Alaska [15]. Lastly, based on the range of δ^15^N and δ^13^C values it is possible that marine mammal consumption is infrequent or that these marine influences are likely from lower trophic prey, such as shorebirds or eggs [103, 124, 126]. For example, δ^15^N and δ^13^C values of this study do not reflect those found in the muscle of ringed seal (range δ^15^N of 13.4–17.5‰, and δ^13^C of -22.2 to -15.0‰), muscle of gulls (range δ^15^N of 16.1–18.0‰, and range δ^13^C of -20.6 to -18.4‰), and salmon carcasses (mean δ^15^N of 12.1 and mean δ^13^C of -19.9‰) [11, 34, 103, 122, 126]. Because the δ^15^N values of wolverine (≤12.9‰) are lower than those marine animals, it is unlikely that marine scavenging is a dominant part of their diet. Nevertheless, infrequent marine resource consumption could lead to higher Hg concentrations in tissues due to the slow elimination of Hg. Our study, which addresses a knowledge gap identified by Glass et al. [52], suggests that cross-ecosystem utilization of food resources may contribute to increased Hg exposure, especially in Arctic coastal populations.

### Influence of landscape factors on THg accumulation

An influence of wet Hg deposition on spatial patterns of THg in wolverine muscle was found (as indicated by a positive correlation) but this variable was less important than diet. The lack of relationship between net Hg deposition and THg in wolverine was unexpected. Net Hg deposition was positively correlated with mean THg concentrations in fur of adult little brown bats (*Myotis lucifugus*) in a broad-scale study across eastern and southern Canada [60]. Here we included muscle samples limited to Arctic and boreal biomes in western Canada, where the fate of net Hg deposition may be driven by different factors than in temperate regions [127]. Furthermore, net Hg deposition models incorporate wet and dry Hg fluxes but challenges still exist in the quantification and simulation of dry Hg deposition, including from AMDEs [65, 127, 128]. In contrast, the moderate-positive correlation of THg in wolverine muscle with wet Hg deposition is consistent with other research [129, 130]. Atmospheric Hg wet deposition reflects mainly precipitation inputs (e.g., rain, snow, etc.) [65, 131]. Yet, non-precipitation Hg wet deposition (cloud, fog, dew, frost, etc.), not considered in the model, may also contribute to deposition patterns in Arctic region [65]. Uncertainties in Arctic atmospheric deposition may be a limiting factor in assessing its relationship with wolverine measurements. Interestingly, higher wet Hg deposition rates were found in Arctic coastal areas (Fig 7) and it covaried with distance to the coast and other climate and landscape variables (**Figs S5 and S6 in** S1 File). The lower explanatory power of wet Hg deposition may be hidden by the effects of local or regional scale processes (e.g., biochemical processes in snow-ice, SOC, land cover type) and feeding ecology.

The landscape variables that explained THg variation in wolverines (% of wet area, SOC and % of snow/ice) may reflect a combination of processes including Hg transport and production of MeHg. The relative importance of wet area % as the second most important explanatory variable of THg concentration in wolverines is consistent with the importance of freshwater bodies and wetlands as hot spots of Hg methylation [5]. Terrestrial wildlife at risk of greater Hg exposure have been linked to aquatic life stages or foraging habits in these wet areas (e.g., from arthropods to birds) [132]. Conversely, aquatic environments (e.g., surface streams and wetlands), can receive significant loads of Hg from terrestrial inputs, such as Hg stored in soils [21]. Globally, terrestrial environments contain the largest reservoir of Hg, mainly stored in organic soils, including permafrost and glacial ice in Arctic regions [19, 133]. The selection of SOC in both models (collection location and 150 km buffer) supports the suggestion that organic matter (OM) plays an important role in Hg accumulation in terrestrial ecosystems. In small terrestrial mammals, soil OM was an important variable explaining THg in hair and in soil of collection sites [134]. In soils, Hg is bound strongly to OM, which can be moved with water throughout terrestrial environments [21]. Recent discoveries have highlighted that Hg methylation is not restricted to wetlands or flooded areas, for example methylation has been documented in forest soils, snow, sea ice, and seawater [8, 21, 135]. Some have argued that MeHg found in terrestrial forest floors could be generated during OM decomposition processes (by fungi and/or bacteria) [135]. In the Arctic, % of organic carbon was also associated with THg concentration in soils. Finally, the Hg cycle of snow and ice is less understood, but thawing of glaciers, permafrost and snowpack can export high yields of Hg from Arctic terrestrial landscapes to receiving watersheds [21]. This mobilized Hg could enter food webs, for example snowfall has significantly predicted Hg in Arctic char (*Salvelinus alpinus*) in an Arctic lake in NU [136]. Given the collinearity of explanatory variables, additional research is needed to evaluate underlying mechanisms of landscape processes.

## Conclusions

Our study revealed broad spatial patterns of THg bioaccumulation in a terrestrial carnivore across Arctic and boreal biomes in western Canada. The highest THg concentrations in wolverine muscle were found near the Arctic coast while the lowest THg burdens were in inland boreal biomes. Diet and surrounding environmental and landscape features played an important role in explaining THg concentrations in wolverines. Enhanced THg bioaccumulation in coastal areas may reflect dietary inputs from marine resources as well as enhanced environmental exposure from atmospheric Hg deposition or greater terrestrial MeHg production. Taken together, our unique dataset for wolverines addresses a data gap on broad-scale drivers of Hg accumulation in terrestrial wildlife. However, further research is needed to identify dominant methylation zones and pathways for Hg uptake into high latitude terrestrial food webs.

## Supporting information

S1 File Appendix. This file contains supplementary text, tables and figures on data sources, methods and statistical results.
(DOCX)

S2 File Raw data on wolverine hair. This file contains data on hydrogen stable isotope ratios of wolverine hair, associated collection information, and environmental variables of collection sites.
(XLSX)

S3 File Raw data on wolverine muscle. This file contains data on mercury concentrations and carbon and nitrogen stables isotope ratios of wolverine muscle, associated collection information, and environmental variables of collection sites.
(XLSX)
