# Supplementary material for: Diet and landscape characteristics drive spatial patterns of mercury accumulation in a high-latitude terrestrial carnivore
Source: PLoS One. 2023 May 15;18(5):e0285826. doi: 10.1371/journal.pone.0285826 (PMC10184919; doi:10.1371/journal.pone.0285826)
Supplement: S1 File — This file contains supplementary text, tables and figures on data sources, methods and statistical results. (DOCX) [file pone.0285826.s001.docx]

**Supporting information for:**

**Diet and landscape characteristics drive spatial patterns of mercury accumulation in a high-latitude terrestrial carnivore**

Inés Peraza^1^, John Chételat*^2^, Murray Richardson^1^, Thomas S. Jung^3,4^, Malik Awan^5^, Steve Baryluk^6^, Ashu Dastoor^7^, William Harrower^8^, Piia M. Kukka^3^, Christine McClelland^2^, Garth Mowat^9,10^, Nicolas Pelletier^2^, Christine Rodford^2^, Andrei Ryjkov^7^

^1^ Geography and Environmental Studies, Carleton University, Ottawa, Ontario K1S 5B6, Canada

^2^ Environment and Climate Change Canada, National Wildlife Research Centre, Ottawa, Ontario K1A 0H3, Canada

^3^ Department of Environment, Government of Yukon, Whitehorse, Yukon Y1A 2C6, Canada

^4^ Department of Renewable Resources, University of Alberta, Edmonton, Alberta, Canada

^5^ Department of Environment, Government of Nunavut, Igloolik, Nunavut, Canada

^6^ Environment and Natural Resources, Government of the Northwest Territories, Inuvik, Northwest Territories, Canada

^7^ Environment and Climate Change Canada, Air Quality Research Division, Dorval, Quebec H9P 1J3, Canada

^8^ Forest and Conservation Sciences, University of British Columbia, Vancouver, British Columbia, Canada

^9^ Ministry of Forests, British Columbia Government, Nelson, British Columbia, Canada

^10^ Department of Earth, Environmental and Geographic Sciences, University of British Columbia, Kelowna, British Columbia, Canada

*Corresponding author

Email: [john.chetelat@ec.gc.ca](mailto:john.chetelat@ec.gc.ca)

Table of Contents

[Text S1. Supplemental information on the data analysis 3](#_Toc128480146)

[Table S1. GIS data and sources used for this research. 7](#_Toc128480147)

[Table S2. The 18 potential model parameters calculated from GIS data and calculations used if required. 10](#_Toc128480148)

[Figure S1. Boxplots showing variation in hair δD values of wolverines across boreal and Arctic biomes in Canada (n = 80). 11](#_Toc128480149)

[Figure S2. Spearman correlation coefficients between δD in wolverine hair and environmental factors collected at the collection location. 12](#_Toc128480150)

[Figure S3. Spearman correlation coefficients between δD in wolverine hair and environmental factors. 13](#_Toc128480151)

[Figure S4. Boxplots showing variation in THg concentration of wolverines in the western Canadian Arctic (n = 419). 14](#_Toc128480152)

[Table S3. Mean, median and range values of THg (μg/g dw) in wolverine muscle, grouped by sex and age (n = 418). 15](#_Toc128480153)

[Figure S5. Spearman correlation coefficients between THg in wolverine muscle and landscape, climate and dietary variables. 16](#_Toc128480154)

[Figure S6. Spearman correlation between THg in wolverine muscle and landscape, climate and dietary variables. 17](#_Toc128480155)

[Figure S7. Climate and elevation datasets used as input for the THg analyses. 18](#_Toc128480156)

[Figure S8. Soil and landcover datasets used as input for the THg analyses. 19](#_Toc128480157)

[Table S4. Mercury concentrations in muscle of selected marine and terrestrial animals. Literature-derived values of THg concentrations (μg/g) from Arctic and non-Arctic terrestrial wildlife in North America and Europe. 20](#_Toc128480158)

[Text S2. Testing of underlying assumptions for the linear regression models 23](#_Toc128480159)

[References 28](#_Toc128480160)

# **Text S1.** Supplemental information on the data analysis

Explanation of GIS data collection and transformations

*Wolverine buffers*

Around each wolverine collection location, a 150 km buffer was created. The buffer size represents a moving distance range that the wolverines could be travelling around the collection location. The 150 km was chosen from the correlation and regression analyses of $\delta$D_h_ and environmental variables. The simple features (sf) package [1] in R [2] was used to create the circular buffers. The layer was projected to Canada Albers Equal Area Conic. These buffers were used to extract summary statistics or clip other GIS layers.

*Ecological Regions of North America, level I and II*

The level I and II North American ecological regions shapefiles [3] were used to group and compare $\delta$D_h_ and THg levels in wolverines. This dataset was obtained from the CEC with the Lambert Azimuthal Equal Area projection. The most updated version (as of July 2021) was acquired. For Canada and the US, the dataset is originated/updated by the U.S. Environmental Protection Agency- National Health and Environmental Effects Research Laboratory (NHEERL) and the Canadian Council on Ecological Areas (CCEA) respectively. The level II ecoregions provide a more detailed description of large ecological areas than the coarser level I ecoregions [4]; thus, they are useful for national/regional perspectives of ecological patterns. The ecoregions delineation follows a holistic approach by integrating the analysis of the patterns and the composition of both living and nonliving phenomena, such as geology, physiography, vegetation, climate, soils, land use, wildlife, hydrology and human factors [3,4].

**Raster data collected**

The following raster datasets represent landscape and climate variables used in this study and were analyzed in R [2] unless indicated otherwise. Spatial transformations and summary statistics were performed using the raster, exactextractr and sf R packages [1,5,6]. Two measurements were calculated for each variable, the pixel value at the collection location and mean pixel value within the 150 km buffer around each wolverine collection location. At the collection location, the nearest cell value to the point location was used if no pixel was available

*Mercury Deposition*

Net and wet Hg deposition for Canada was modelled using the operational air quality forecast model GEM-MACH-Hg (Global Environmental Multi-scale, Modelling Air quality and CHemistry model). Two interpolated raster files were supplied, net (HgDep) and wet (HgWet), averaging annual Hg deposition rates (μg/m^2^) for 2015. The files had a spatial resolution of 0.03 x 0.02 degrees (longitude, latitude). These files were reprojected into Canada Alber's equal-area conic projection, resulting in a 1650 x 2260 m raster cell size.

*Precipitation and Temperature*

Precipitation accumulation (mm) and maximum and minimum temperature (°C) raster files were retrieved from TerraClimate [7] using the cilmateR package [8] in R. TerraClimate provides monthly temporal resolution data (from 1958-present) and a ~4-km (2.5 arc-minutes) spatial resolution [7,9]. The climate datasets downloaded correspond to the range of years from the trapping seasons of the wolverines (2005-2018); a boundary for Canada-USA was used to spatially subset the datasets. The raster files were projected to North America Albers equal-area conic projection because some of the wolverines are neighbouring Alaska border. The trapping season of each wolverine was used to summarize the mean climate variables. That is, if the wolverine was trapped in 2006-2007, the mean precipitation and temperatures were estimated for those years.

*Elevation*

A digital surface model (DSM) raster dataset was accessed through Google Earth Engine (GEE) [10] using the rgee package [11] in R. The dataset used (JAXA/ALOS/AW3D30/V3_2) is provided by the Earth Observation Research Center (EORC) of the Japan Aerospace Exploration Agency (JAXA) has an approximate 30 m spatial resolution and is based on the 5 m DSM dataset of the World 3D Topographic Data [12,13]. The extracted values were exported from GEE to a local drive.

*Soil Organic Carbon (SOC)*

A raster file of SOC stock (t/ha) from 0 to 30 cm depth was obtained from the Global Soil Organic Carbon Map web service [14]. The file had a spatial resolution of 0.5 arc-minutes (~ 1 km) [15]. The file was spatially subset using a boundary for Canada-Alaska and reprojected to Canada Albers equal-area conic projection.

*Soil pH (spH)*

A soil pH, measured in soil-water solution, raster dataset was accessed through GEE using the rgee package[11] in R. The dataset used (OpenLandMap/SOL/SOL_PH-H2O_USDA-4C1A2A_M/v02) has a 250 m spatial resolution and averages the 1950-2018 period [16]. Measurements from two image bands were collected, soil pH at 10 cm depth (topsoil) and 60 cm depth (subsoil). The extracted values were exported from GEE to a local drive.

*Landcover*

The 2015 North American Land Cover (NALC) 30-meter dataset [17] was used to determine the area of forest, shrubland, grassland, barren land, wetland, open water, and snow-ice within the 150 km buffer. The NALC had the Lambert Azimuthal Equal Area projection and was provided by the Commission for Environmental Cooperation (CEC) through the North American Environmental Atlas ([www.cec.org/north-american-environmental-atlas/](http://www.cec.org/north-american-environmental-atlas/)). The Tabulate Area tool from the Spatial Analyst (Zonal) in ArcGIS Pro 2.8.2 [18] was used to calculate the area of each land cover class within each buffer around the wolverine collection location.

The measures of forest land (NALC value 1 = Temperate or sub-polar needle leaf forest, value 2 = Sub-polar taiga needle leaf forest, value 5 = Temperate or sub-polar broadleaf deciduous forest, and value 6 = Mixed Forest) were summed together to determine the total area of forested land within each buffer. Similarly, the following landcover classes were summed together to determine the total area of shrubland, grassland, and barren land within each buffer. The two measures of shrubland (NALC value 8 = Temperate or sub-polar shrubland and value 11 = Sub-polar or polar shrubland-lichen-moss); the two measures of grassland (NALC value 10 = Temperate or sub-polar grassland and value 12 = Sub-polar or polar grassland-lichen-moss); the two measurements of barren land (NALC value 13 = Sub-polar or polar barren-lichen-moss and value 16 = Barren lands). NALC value 14= Wetland, value 18 = Water and value 19 = Snow and ice, were maintained as independent landcover classes.

Table S1. GIS data and sources used for this research.

| **Grid / Layer** | **Format / CRS** | **Spatial Resolution (if grid)** | **Description** | **Sources** |
| --- | --- | --- | --- | --- |
| Net and wet Hg deposition | Geo Tiff / WGS84 | x:1.8; y: 1.2 arc-minutes (~ 1.6 x 2.2 km) | Net and wet Hg deposition rates to terrestrial and aquatic ecosystems. Interpolated files from the original model with 1° x 1° of spatial resolution. | A. Dastoor & A. Ryjkov, |
| Precipitation | Geo Tiff / WGS84 | 2.5 arc-minutes (~ 4 km) | Monthly amount of precipitation accumulation (mm). | [7,9] |
| Temperature | Geo Tiff / WGS84 | 2.5 arc-minutes (~ 4 km) | Monthly minimum temperature and maximum temperature (°C). | [7,9] |
| Land cover | Geo Tiff / Lambert Azimuthal Equal Area | 30 m | North American land cover (NALC), version 2 provides a standardized landcover dataset across the U.S.A, Canada and Mexico based on 2015 Landsat satellite. An updated land cover map of Alaska was provided in July 2020 by the United States Geological Survey (USGS). | [17] |
| Elevation | ee.ImageCollection / WGS84 | 30 m | The ALOS World 3D - 30m (AW3D30) is a DSM derived from the Panchromatic Remote-sensing Instrument for Stereo Mapping (PRISM), optical sensor on board the Advanced Land Observing Satellite "ALOS". The latest version has been updated to improve the absolute/relative height accuracies with additional calibrations from other open-access DSMs (e.g., Shuttle Radar Topography Mission (SRTM) Digital Elevation Model (DEM), Advanced Spaceborne Thermal Emission and Reflection Radiometer Global DEM (ASTER GDEM), ArcticDEM) | [10,12,13] |
| Soil Organic Carbon | Geo Tiff / WGS84 | 0.5 arc-minutes (~ 1 km) | Soil organic carbon stock from 0 to 30 cm, tonnes/ha. | [14,15] |
| Soil pH | ee.Image / WGS84 | 250 m | Global soil pH in H2O at 6 standard depths (0, 10, 30, 60, 100 and 200 cm) from 1950 to 2018. | [10,16] |
| Canada and USA boundaries | Shapefile / WGS84 | N/A | Sovereign states (Admin - 0) polygons. Scale 1:10m; version 4.1.0. Canada and USA boundaries were subset from the original file and converted to polylines. | [19] |
| NA Ecozones boundaries | Shapefile / Lambert Azimuthal Equal Area | N/A | This data set includes ecological regions for all of North America and represents an integration of the most updated datasets released by the governments of the United States of America, Mexico and Canada. Last update was in Aug 2020-Jun 2021. | [3,4] |

Landscape and climate variables calculations

*Distance to Arctic coast calculation*

For each wolverine collection location point, the shortest distance to the Arctic coastline was calculated in meters. The boundary line vector of Canada and the USA was used as the coastline reference [19]. The sf R package was used to calculate the distance between each wolverine location point and the nearest boundary line. The North America equidistant conic projection was applied to both point and line features to ensure appropriate calculation of distances [20].

Table S2. The 18 potential model parameters calculated from GIS data and calculations used if required.

| **Variable** | **Abbreviation** | **Calculation** |
| --- | --- | --- |
| Net Hg deposition | HgDep | Mean pixel value within 150 km buffer |
| Wet Hg deposition | HgWet |  |
| Mean precipitation | *prcp* |  |
| Mean maximum temperature | *Tmax* |  |
| Mean minimum temperature | *Tmin* |  |
| Mean elevation | Elev |  |
| Distance to Arctic coast | DistCoast | The shortest distance from the collection location to the coastline, in meters. The boundary line vector of the Arctic Ocean and Hudson Bay in Canada was used as the coastline |
| Mean soil organic carbon | SOC | Mean pixel value within 150 km buffer |
| Mean subsoil pH | spH60 |  |
| Mean topsoil pH | spH10 |  |
| Forest % | Forest % | NALC Forest / Buffer area * 100 |
| Shrubland % | Shrubland % | NALC Shrubland / Buffer area * 100 |
| Grassland % | Grassland % | NALC Grassland / Buffer area * 100 |
| Barren land % | Barren land % | NALC Barren Land / Buffer area * 100 |
| Wetland % | Wetland % | NALC Wetland / Buffer area * 100 |
| Water % | Water % | NALC Water Land/ Buffer area * 100 |
| Snow-ice % | Snow-ice % | NALC Snow & Ice Land / Buffer area * 100 |
| Wet Area % | Wet Area % | NALC Wetland + Water Land / Buffer area * 100 |


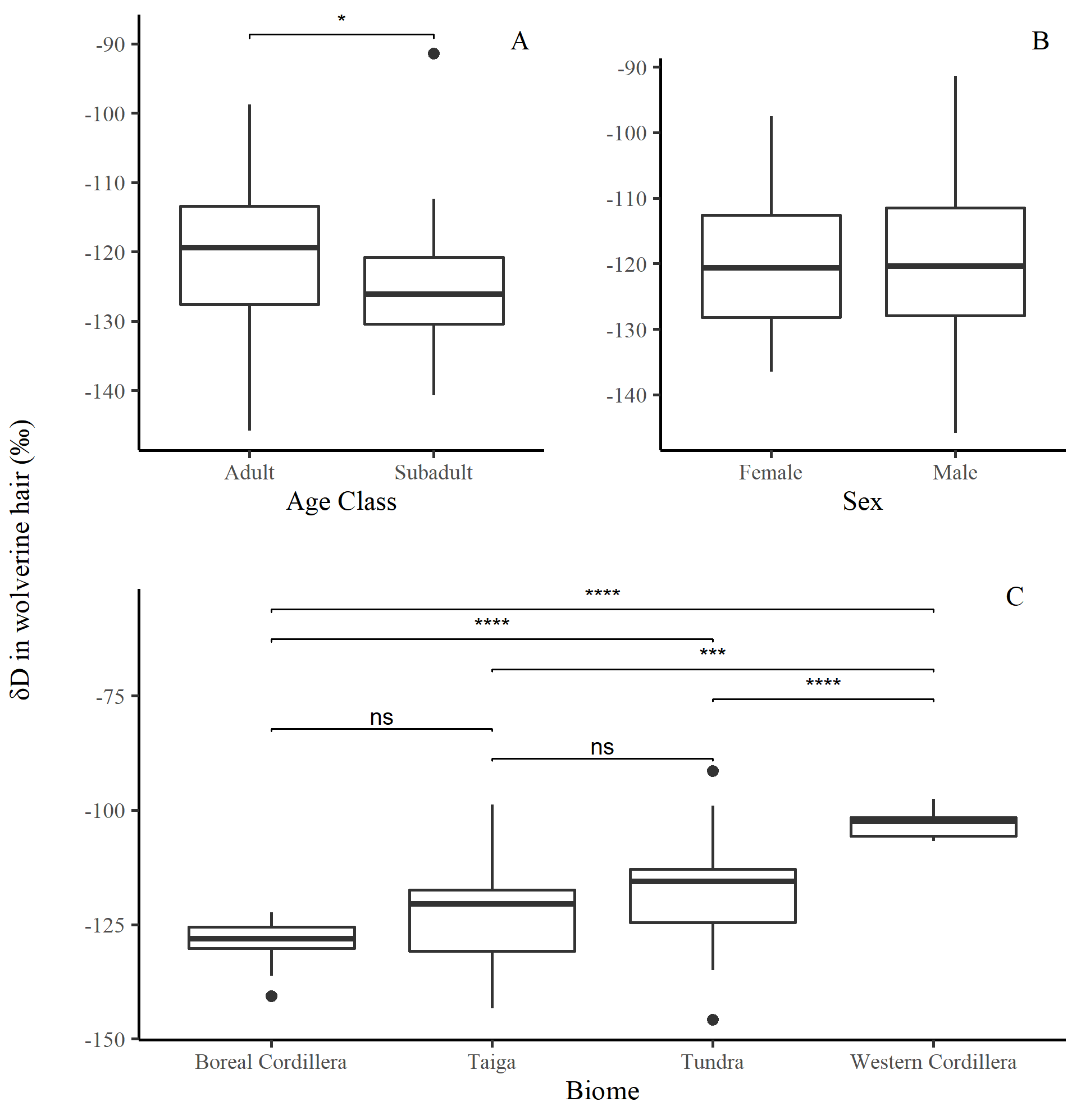


Figure S1. Boxplots showing variation in hair δD values of wolverines across boreal and Arctic biomes in Canada (n = 80).

A: Adult (> 2 year old) and subadult (≤2 year old). ns: p> 0.05, ⋆: p≤ 0.05, ⋆⋆: p≤ 0.01, ⋆⋆⋆: p≤ 0.001, ⋆⋆⋆⋆: p≤ 0.0001.


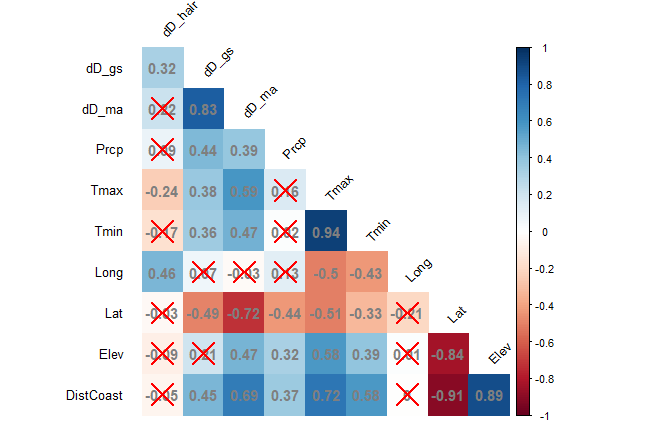


Figure S2. Spearman correlation coefficients between δD in wolverine hair and environmental factors collected at the collection location.

The variable names are shown on the left and upper diagonal. Rho values are displayed inside the coloured squares. Positive correlations are represented in blue and negative correlations are in red. Correlations with p-value > 0.05 were considered not significant (crossed squares).


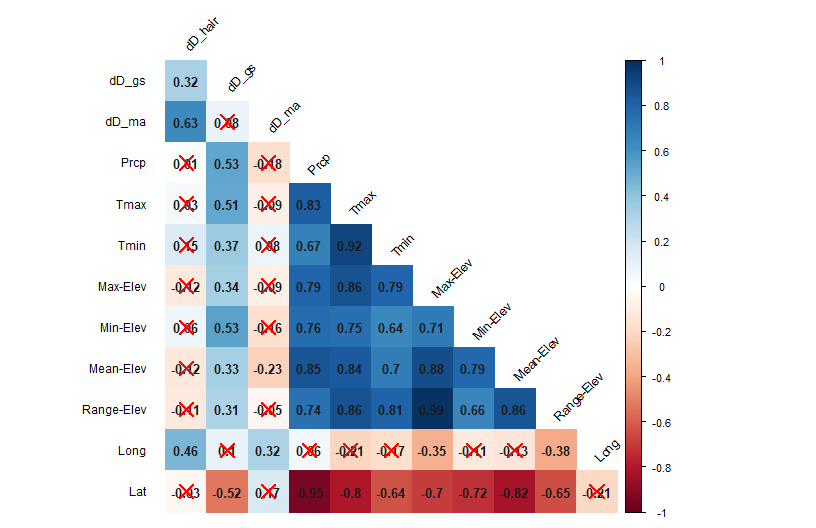


Figure S3. Spearman correlation coefficients between δD in wolverine hair and environmental factors.

Mean values were collected around a 150 km radius from the collection location. Variable names, rho values and significance levels as described in Figure S2.


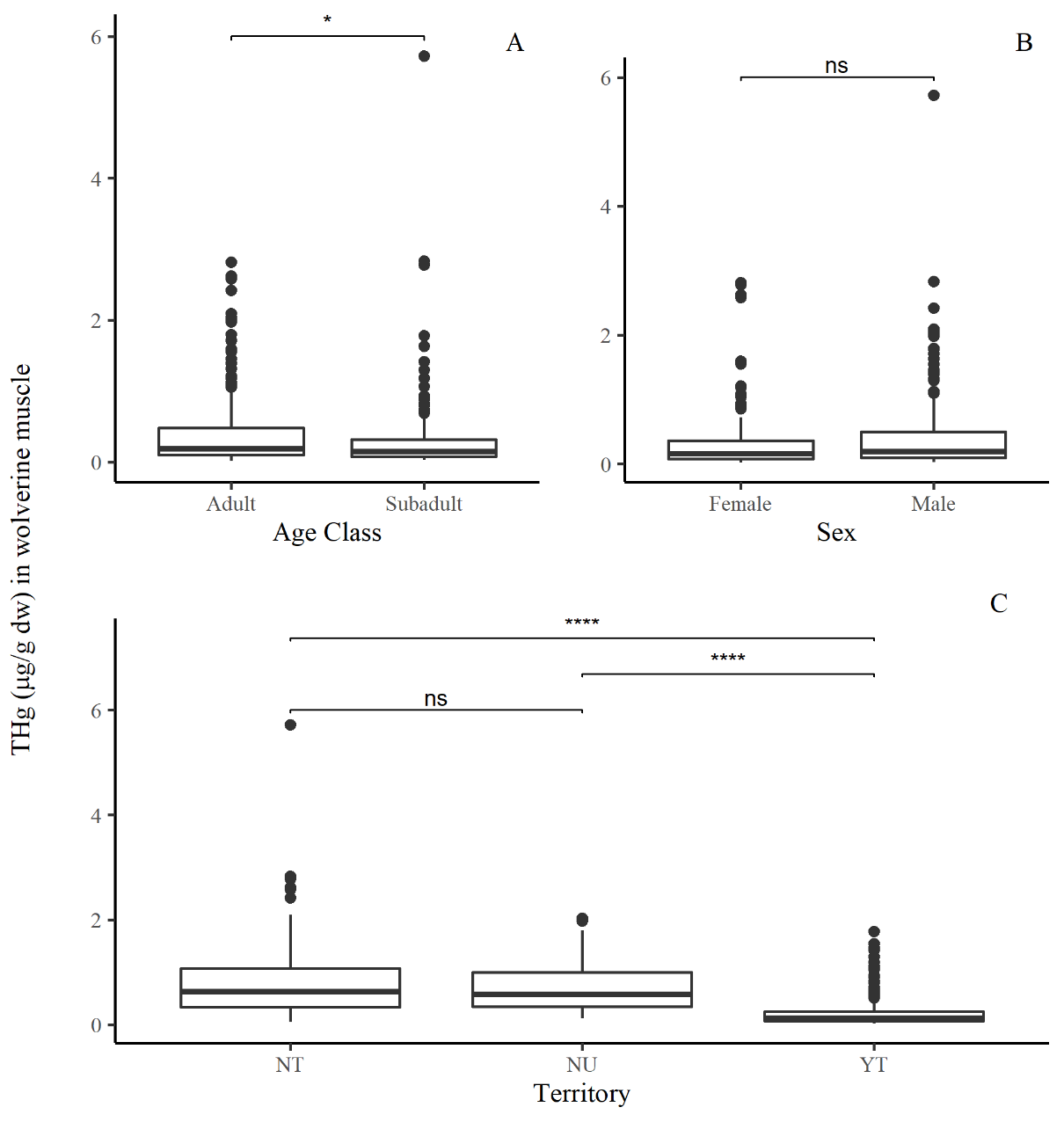


Figure S4. Boxplots showing variation in THg concentration of wolverines in the western Canadian Arctic (n = 419).

Plot A: N = 411 and plot B: n = 418, due to unknown records. Age classes and significance levels as in Figure S1.

Table S3. Mean, median and range values of THg (μg/g dw) in wolverine muscle, grouped by sex and age (n = 418).

|  | **Female** | | | **Male** | | |
| --- | --- | --- | --- | --- | --- | --- |
| **THg** | **Adult**  **N = 92** | **Sub-adult**  **N = 53** | **Unknown**  **N = 2** | **Adult**  **N = 162** | **Sub-adult**  **N = 104** | **Unknown**  **N = 5** |
| Mean  (SD) | 0.38  (0.55) | 0.28  (0.44) | 0.21  (0.08) | 0.40  (0.47) | 0.37  (0.68) | 0.52  (0.60) |
| Median | 0.16 | 0.13 | 0.21 | 0.22 | 0.16 | 0.38 |
| Range | 0.01,  2.82 | 0.03,  2.78 | 0.15,  0.26 | 0.03,  2.42 | 0.03,  5.72 | 0.09,  1.54 |

* One subadult was of unknown sex with a THg concentration of 0.32 (µg/g dw)


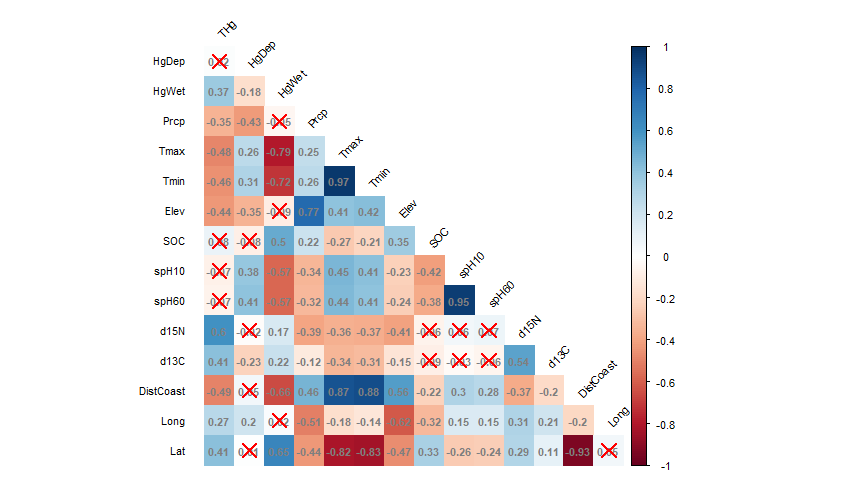


Figure S5. Spearman correlation coefficients between THg in wolverine muscle and landscape, climate and dietary variables.

Values collected at the collection location. Variable names, rho values and significance levels as described in Figure S2.


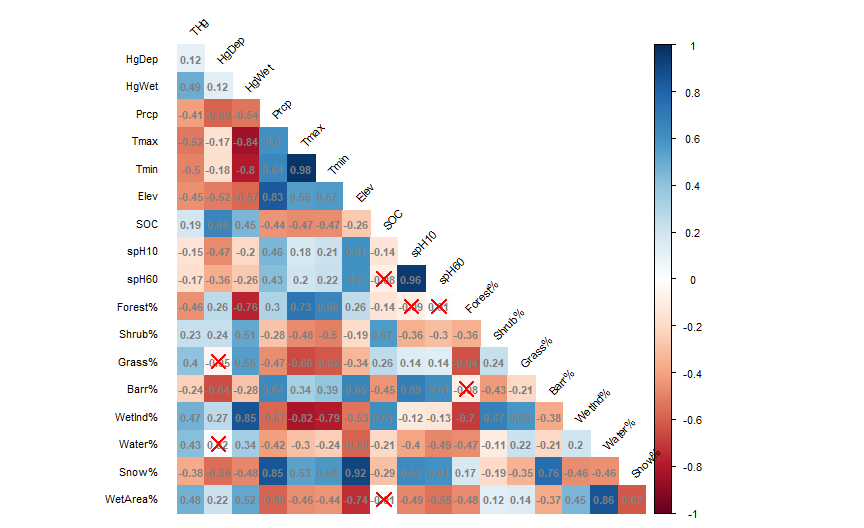


Figure S6. Spearman correlation between THg in wolverine muscle and landscape, climate and dietary variables.

Mean values were collected around a 150 km radius from the collection location. Variable names, rho values and significance levels as described in Figure S2.


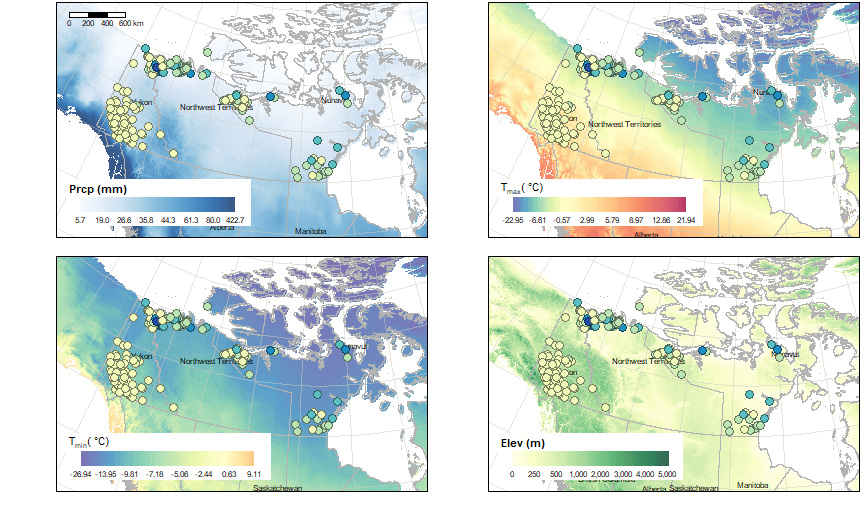
Figure S7. Climate and elevation datasets used as input for the THg analyses.

Circles represent muscle THg concentrations in wolverines (µg/g dw).


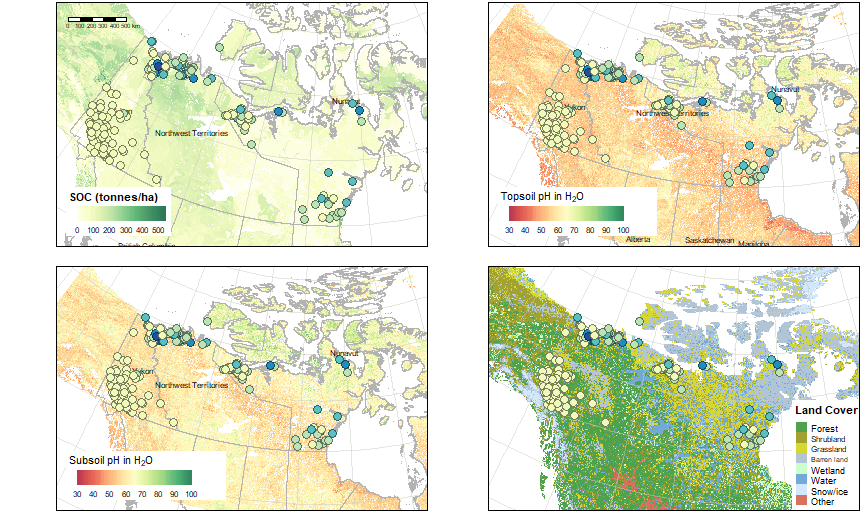
Figure S8. Soil and landcover datasets used as input for the THg analyses.

Circles represent muscle THg concentrations in wolverines (μg/g dw). Soil pH datasets scaled x10.

Table S4. Mercury concentrations in muscle of selected marine and terrestrial animals. Literature-derived values of THg concentrations (μg/g) from Arctic and non-Arctic terrestrial wildlife in North America and Europe.

| **Species** | **THg** | | **Measurement** | **Location** | **Source** |
| --- | --- | --- | --- | --- | --- |
|  | **dw** | **ww*** |  |  |  |
| Wolverine | 0.17, 0.37  (0.01 - 5.72) |  | M, AM  (Min-Max) | Canadian Arctic | This study |
| Polar bear | ~0.136 – 0.764 | 0.034 – 0.191 | GM Min – GM Max/loc | Northwest & east Greenland | [21] |
|  | 0.68  (0.2 – 1.8) |  | AM  (Min-Max) | NU, Canada | [22] |
|  | 0.84 |  | AM | Lancaster Sound, NT | [23] |
| Beluga whale | ~10.4 | 2.6 | AM | Great Whale, Quebec | [24,25] |
| Ringed seal | ~0.4 - 4.28 | 0.10 - 1.07 | AM Min - AM Max/loc | Canadian Arctic | [26] |
|  | ~0.2 - 3.56 | 0.05 - 0.89 |  |  | [27] |
| Gray wolf | ~0.05, ~0.328  (~0.01 - 2.18) | 0.0125, 0.082  (0.0044 – 0.546) | M, AM  (Min-Max) | Alaska | [28,29] |
|  | ~0.03 | 0.00671 | AM | Dinaric Alps, Croatia | [30] |
| Red fox | 0.06  (0.02-0.23) |  | GM  (Min-Max) | North-Western Poland | [31] |
|  | 0.05, 0.07  (0.02 – 0.24) |  | M, AM  (Min-Max) |  | [32] |
|  | ~0.08 | <0.02 | AM | Wisconsin, US | [33] |
| Brown bear | ~0.016 | 0.00411 | AM | Dinaric Alps, Croatia | [30] |
| Mink | ~8.55 | ~2.14 | AM | James Bay, Quebec | [34] |
| Mink | ~2.23-5.44 | 0.558-1.36 | AM Min - AM Max/loc | Rhode Island (USA) | [35] |
| Otter | ~1.28 - 3.08 | 0.32 - 0.77 | GM Min – GM Max/loc | Wisconsin, US | [36] |
|  | ~5.15 | ~1.29 | AM | James Bay, Quebec | [34] |
| Marten | ~1.12 | 0.28 | AM | Great Whale (Hudson Bay) | [24] |
| Snowshoe hare | ~0.2 | <0.05 | AM | Great Whale (Hudson Bay) | [24] |
| Muskrat | ~0.08 | <0.02 | AM | Wisconsin, US | [33] |
| Beaver | ~0.08 | <0.02 | AM | Wisconsin, US | [33] |
| Caribou | ~0.08 - 0.12 | 0.019 – 0.030 | AM Min - AM Max/loc | Northern Québec | [37] |
|  | 0.02- 0.05 |  | AM Min - AM Max/loc | West Greenland | [38] |
|  | ~0.02 | 0.005 | AM | Mackenzie Mountains, NT | [39] |
| Moose | ~0.008 | <0.002 | AM | Mackenzie Mountains, NT | [39] |
|  | ~0.016  ~(0.004 – 0.156) | 0.004  <0.001-0.039 | AM  (Min-Max) | Northwest Russia | [40] |
| Waterfowl  (4 spp. pooled) | 0.88 |  | AM | Northeastern Canada | [41] |
| Coastal gulls  (3 spp. pooled) | 3.42 |  |  |  |  |
| Pelagic seabirds  (6 spp. pooled) | 2.29 |  |  |  |  |

Dry weight (dw) and wet weight (ww). *If conversion from ww into dw was not provided, moisture content of 75% was assumed and the formula from Eccles et al. [42] was applied. If required THg concentrations were converted to μg/g. AM: arithmetic mean, GM: geometric mean, M: median, loc: location.

# **Text S2.** Testing of underlying assumptions for the linear regression models

In all models, independence of independent variables was considered by removing variables that were correlated (e,g, high Spearman rho value). Conversion of independent variables and adding interaction terms were tested to check any changes on model fit and the LM assumptions.

**Table 4 models’ assumptions (2H)**

| **Assumption** | **Collection location** | **150 km buffer** |
| --- | --- | --- |
| **Multivariate Normality – tested with plots (QQ below) and normality tests.**  **(p > 0.05, res are normal)** | Shapiro-Wilk normality test  data: residuals(fit4_sl_sub)  W = 0.98523, **p-value = 0.5436** | Shapiro-Wilk normality test  data: residuals(fitAll150_6_sub)  W = 0.98246, **p-value = 0.3953** |
|  | Kolmogorov-Smirnov test  data: residuals(fit4_sl_sub)  D = 0.090906, **p-value = 0.5433**  alternative hypothesis: two-sided | Kolmogorov-Smirnov test  data: residuals(fitAll150_6_sub)  D = 0.082543, **p-value = 0.6636**  alternative hypothesis: two-sided |
| **No Multicollinearity—tested using Variance Inflation Factor (VIF) values.** | Hma_rcwip = VIF 1.222283  temp_max = VIF 1.222283 | Hma_rcwip150Km = VIF 1.071618  Lat = VIF 2.168784  maxElev_150Km = VIF 2.089670 |
| **Homoscedasticity – see plots below** | Multiple iterations of removal/addition of dependent variables and removing of outliers/leverage were tested. In some cases, removing outliers and/or leverage points did not improve model or decrease the fit. After each iteration and/or removing the outlier and the leverage points the regression assumptions were checked again. Studentized residuals were plotted to help identifying outliers. Studentized residuals were checked with Bonferroni Outlier Tests (p < 0.05). No studentized residual that exceeded +- 3 were kept in the models.  The models that yielded “better” residuals, fit, and VIF were kept. Decided keeping points that seem influential in the model but we could make a careful note about this when reporting the regression results. | |


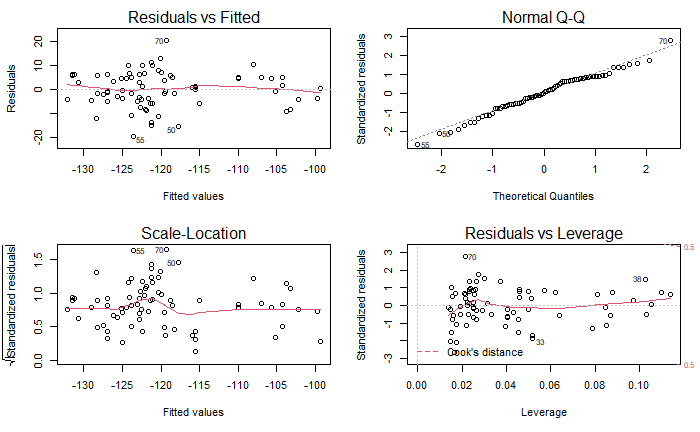


2H Sample location model residuals


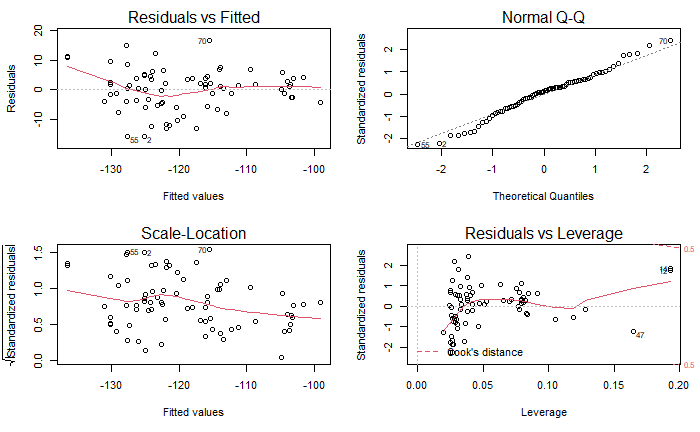


2H – 150 km buffer model residuals

**Table 6 models’ assumptions – ln(THg)**

| **Assumption** | **Collection location** | **150 km buffer** |
| --- | --- | --- |
| **Multivariate Normality – tested with plots (QQ below) and normality tests.**  **(p > 0.05, res are normal)** | Shapiro-Wilk normality test  data: residuals(mod17_site_sub)  W = 0.9941, **p-value = 0.** **1219** | Shapiro-Wilk normality test  data: residuals(mod16_buf2_sub)  W = 0.99382, **p-value = 0.09761** |
|  | Kolmogorov-Smirnov test  data: residuals(mod17_site_sub)  D = 0.035325, **p-value = 0.6974**  alternative hypothesis: two-sided | Kolmogorov-Smirnov test  data: residuals(mod16_buf2_sub)  D = 0.041484, **p-value = 0.487**  alternative hypothesis: two-sided |
| **No Multicollinearity—tested using Variance Inflation Factor (VIF) values.** | soc = VIF 1.010496  coastDist = VIF 1.291055  Delta15N = VIF 1.302856 | soc = VIF 1.242992  Delta15N = VIF 1.252845  snow_perc = VIF 1.309275  wetArea_perc = VIF 1.587995 |
| **Homoscedasticity – see plots below** | Multiple iterations of removal/addition of dependent variables and removing of outliers/leverage were tested. In some cases, removing outliers and/or leverage points did not improve model or decrease the fit. After each iteration and/or removing the outlier and the leverage points the regression assumptions were checked again. Studentized residuals were plotted to help identifying outliers. Studentized residuals were checked with Bonferroni Outlier Tests (p < 0.05). No studentized residual that exceeded +- 3 were kept in the models.  The models that yielded “better” residuals, fit, and VIF were kept. | |


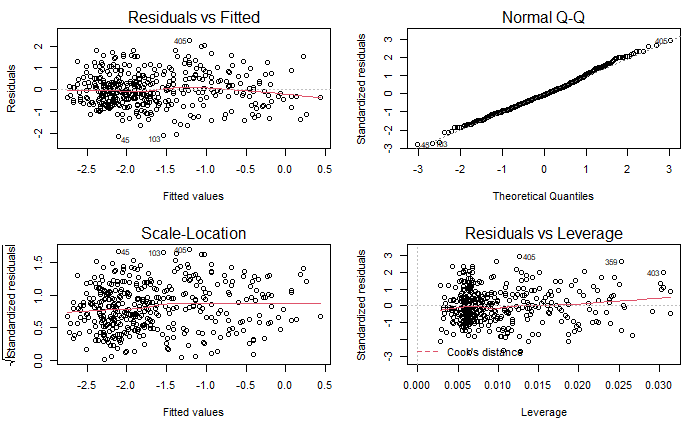
ln(THg) Sample location model residuals


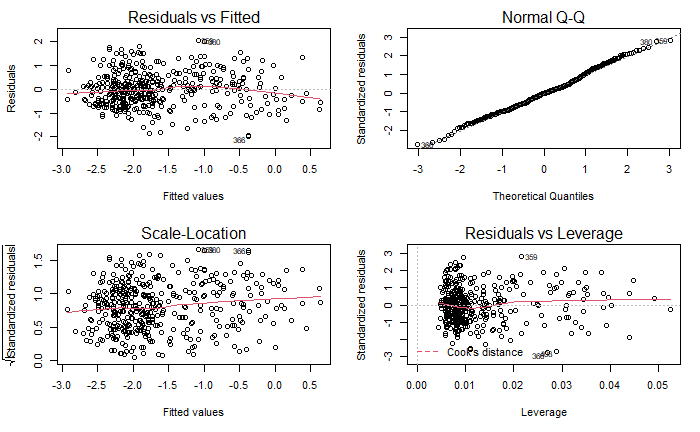


ln(THg) – 150 km buffer model residuals

# **References**

1. Pebesma E. sf: Simple Features for R [Internet]. 2021. Available from: https://cran.r-project.org/package=sf

2. R Core Team. R: A Language and Environment for Statistical Computing [Internet]. 2020;Available from: https://www.r-project.org

3. CEC. Terrestrial Ecoregions: Level II. Version 2 [Vector digital data] [Internet]. N. Am. Environ. Atlas2021;Available from: http://www.cec.org/north-american-environmental-atlas/terrestrial-ecoregions-level-ii/

4. CEC. Ecological Regions of North America: Toward a Common Perspective [Internet]. Montréal, Québec, Canada: Commission for Environmental Cooperation; 1997. Available from: http://www.cec.org/files/documents/publications/1701-ecological-regions-north-america-toward-common-perspective-en.pdf

5. Baston D. exactextractr: Fast Extraction from Raster Datasets using Polygons [Internet]. 2021. Available from: https://cran.r-project.org/package=exactextractr

6. Hijmans RJ. raster: Geographic Data Analysis and Modeling [Internet]. 2021. Available from: https://rspatial.org/raster

7. University of California Merced. TerraClimate [Internet]. Climatol. Lab [cited 2021 Feb 21];Available from: http://www.climatologylab.org/terraclimate.html

8. Johnson M. climateR: An R client for climate data [Internet]. 2021. Available from: https://github.com/mikejohnson51/climateR

9. Abatzoglou JT, Dobrowski SZ, Parks SA, Hegewisch KC. TerraClimate, a high-resolution global dataset of monthly climate and climatic water balance from 1958-2015. Sci. Data 2018;5:1–12.

10. Gorelick N, Hancher M, Dixon M, Ilyushchenko S, Thau D, Moore R. Google Earth Engine: Planetary-scale geospatial analysis for everyone. Remote Sens. Environ. 2017;202:18–27.

11. Aybar C, Wu Q, Bautista L, Yali R, Barja A. rgee: An R package for interacting with Google Earth Engine. J. Open Source Softw. 2020;5:2272.

12. JAXA EORC. ALOS Global Digital Surface Model (DSM) “ALOS World 3D-30m” (AW3D30) Version 3.2/3.1. Product Description [Internet]. Edition 1. Available at Earth Engine Data Catalog; 2021. Available from: https://www.eorc.jaxa.jp/ALOS/en/aw3d30/aw3d30v3.2{\_}product{\_}e{\_}e1.2.pdf

13. Takaku J, Tadono T, Doutsu M, Ohgushi F, Kai H. Updates of ‘AW3D30’ ALOS Global Digital Surface Model with Other Open Access Datasets. Int. Arch. Photogramm. Remote Sens. Spat. Inf. Sci. 2020;XLIII-B4-2:183–9.

14. FAO, ITPS. GLOSIS - GSOCmap v1.5.0 [Internet]. Glob. Soil Org. Carbon Map2019 [cited 2021 Sep 1];Available from: http://54.229.242.119/GSOCmap/#

15. FAO, ITPS. Global Soil Organic Carbon Map V1.5: Technical Report [Internet]. Rome: Food and Agriculture Organization of the United Nations; 2020. Available from: https://doi.org/10.4060/ca7597en

16. Hengl T. Soil pH in H2O at 6 standard depths (0, 10, 30, 60, 100 and 200 cm) at 250 m resolution (Version v0.2) [Data set] [Internet]. Zenodo2018;Available from: https://zenodo.org/record/2525664

17. Natural Resources Canada, Comisión Nacional para el Conocimiento y Uso de la Biodiversidad, Comisión Nacional Forestal, Instituto Nacional de Estadística y Geografía, U.S. Geological Survey. 2015 Land Cover of North America at 30 meters [Internet]. Raster Digit. Data2020 [cited 2021 Sep 10];Available from: http://www.cec.org/north-american-environmental-atlas/land-cover-30m-2015-landsat-and-rapideye/

18. Esri Inc. ArcGIS Pro (Version 2.8.2) [Internet]. 2021;Available from: https://www.esri.com/en-us/arcgis/products/arcgis-pro/overview

19. Natural Earth. Canada and United States of America polygons, using ArcGIS Pro, as a subet of the original dataset [Internet]. 110m Cult. Vectors Admin 0 - Sovereignty Shapefile Geospatial Data Version 410 [cited 2021 May 18];Available from: https://www.naturalearthdata.com/downloads/10m-cultural-vectors/

20. Maher MM. Lining up data in ArcGIS: a guide to map projections. Second edi. Redlands, California: ESRI Press; 2013.

21. Dietz R, Riget F, Born EW. Geographical differences of zinc, cadmium, mercury and selenium in polar bears (Ursus maritimus) from Greenland. Sci. Total Environ. 2000;245:25–47.

22. Bechshoft T, Dyck M, St. Pierre KA, Derocher AE, St. Louis V. The use of hair as a proxy for total and methylmercury burdens in polar bear muscle tissue. Sci. Total Environ. 2019;686:1120–8.

23. Atwell L, Hobson KA, Welch HE. Biomagnification and bioaccumulation of mercury in an arctic marine food web: Insights from stable nitrogen isotope analysis. Can. J. Fish. Aquat. Sci. 1998;55:1114–21.

24. Langlois C, Langis R. Presence of airborne contaminants in the wildife of northern Quebec. Sci. Total Environ. 1995;160–161:391–402.

25. Langlois C, Langis R, Pérusse M. Mercury Contamination in Northern Québec Environment and Wildlife. Water. Air. Soil Pollut. 1995;80:1021–4.

26. Brown TM, Fisk AT, Wang X, Ferguson SH, Young BG, Reimer KJ, et al. Mercury and cadmium in ringed seals in the Canadian Arctic: Influence of location and diet. Sci. Total Environ. 2016;545–546:503–11.

27. Houde M, Taranu ZE, Wang X, Young B, Gagnon P, Ferguson SH, et al. Mercury in Ringed Seals (Pusa hispida) from the Canadian Arctic in Relation to Time and Climate Parameters. Environ. Toxicol. Chem. 2020;39:2462–74.

28. McGrew AK. Spatial Trends of Total Mercury (THg) Exposure, and the Role of Intestinal Helminths on its Distribution within Piscivorous Mammalian Hosts. 2011;

29. McGrew AK, Ballweber LR, Moses SK, Stricker CA, Beckmen KB, Salman MD, et al. Mercury in gray wolves (Canis lupus) in Alaska: Increased exposure through consumption of marine prey. Sci. Total Environ. 2014;468–469:609–13.

30. Lazarus M, Sekovanić A, Orct T, Reljić S, Kusak J, Jurasović J, et al. Apex predatory mammals as bioindicator species in environmental monitoring of elements in Dinaric Alps (Croatia). Environ. Sci. Pollut. Res. Int. 2017;24:23977–91.

31. Kalisinska E, Lisowski P, Salicki W, Kucharska T, Kavetska K. Mercury in wild terrestrial carnivorous mammals from north-western Poland and unusual fish diet of red fox. Acta Theriol. (Warsz.)2009;54:345–56.

32. Kalisinska E, Lisowski P, Kosik-Bogacka, Izabela D, Kosik-Bogacka DI. Red fox Vulpes vulpes (L., 1758) as a bioindicator of mercury contamination in terrestrial ecosystems of north-western poland. Biol. Trace Elem. Res. 2012;145:172–80.

33. Sheffy TB, St. Amant JR. Mercury Burdens in Furbearers in Wisconsin. J. Wildl. Manag. 1982;46:1117–20.

34. Fortin C, Beauchamp G, Dansereau M, Larivière N, Bélanger D. Spatial variation in mercury concentrations in wild mink and river otter carcasses from the James Bay territory, Québec, Canada. Arch. Environ. Contam. Toxicol. 2001;40:121–7.

35. Lake JL, Ryba SA, Serbst J, Brown IV CF, Gibson L. Mercury and stable isotopes of carbon and nitrogen in mink. Environ. Toxicol. Chem. 2007;26:2611–9.

36. Strom SM. Total mercury and methylmercury residues in river otters (Lutra canadensis) from Wisconsin. Arch. Environ. Contam. Toxicol. 2008;54:546–54.

37. Robillard S, Beauchamp G, Paillard G, Bélanger D. Levels of cadmium, lead, mercury and 137caesium in caribou (Rangifer tarandus) tissues from Northern Québec. Arctic 2002;55:1–9.

38. Gamberg M, Cuyler C, Wang X. Contaminants in two West Greenland caribou populations. Sci. Total Environ. 2016;554–555:329–36.

39. Larter NC, Macdonald CR, Elkin BT, Wang X, Harms NJ, Gamberg M, et al. Cadmium and other elements in tissues from four ungulate species from the Mackenzie Mountain region of the Northwest Territories, Canada. Ecotoxicol. Environ. Saf. 2016;132:9–17.

40. Eltsova L, Ivanova E. Total mercury level in tissues of commercial mammalian species (wild boar, moose) of the Russky Sever National Park (North-West of Russia). E3S Web Conf. 2021;265:05009.

41. Mallory ML, Provencher JF, Robertson GJ, Braune BM, Holland ER, Klapstein S, et al. Mercury concentrations in blood, brain and muscle tissues of coastal and pelagic birds from northeastern Canada. Ecotoxicol. Environ. Saf. 2018;157:424–30.

42. Eccles K, Thomas P, Man Chan H. Predictive Meta-Regressions Relating Mercury Tissue Concentrations of Freshwater Piscivorous Mammals. Environ. Toxicol. Chem. 2017;36:2377–84.
